# Long-term physical health conditions and youth anxiety and depression: Is there a causal link?

**DOI:** 10.1017/S0033291724003271

**Published:** 2025-02-04

**Authors:** Amy Shakeshaft, Jessica R. Mundy, Emil M. Pedersen, Charlotte A. Dennison, Lucy Riglin, Daniela Bragantini, Elizabeth C. Corfield, Ajay K. Thapar, Ole A. Andreassen, Evie Stergiakouli, George Davey Smith, Laurie Hannigan, Katherine L. Musliner, Alexandra Havdahl, Anita Thapar

**Affiliations:** 1 Wolfson Centre for Young People’s Mental Health, Division of Psychological Medicine and Clinical Neuroscience, Cardiff University, UK; 2 Centre for Neuropsychiatric Genetics and Genomics, Division of Psychological Medicine and Clinical Neurosciences, Cardiff University, Cardiff, UK; 3 Department for Clinical Medicine, Aarhus University, Denmark; 4 National Centre for Register-based Research, Department of Public Health, Aarhus University, Denmark; 5 Nic Waals Institute, Lovisenberg Diaconal Hospital, Oslo, Norway; 6 PsychGen Center for Genetic Epidemiology and Mental Health, Norwegian Institute of Public Health, Norway; 7 Norwegian Centre for Mental Disorders Research (NORMENT), Institute of Clinical Medicine, University of Oslo and Oslo University Hospital, Norway; 8 Population Health Sciences and MRC Integrative Epidemiology Unit, Bristol Medical School, University of Bristol, UK; 9 PROMENTA Research Centre, Department of Psychology, University of Oslo

**Keywords:** Anxiety, Depression, The Norwegian Mother, Father, and Child Cohort Study, MoBa, Long-term physical health condition, Pediatrics

## Abstract

**Background:**

The prevalence of youth anxiety and depression has increased globally, with limited causal explanations. Long-term physical health conditions (LTCs) affect 20–40% of youth, with rates also rising. LTCs are associated with higher rates of youth depression and anxiety; however, it is uncertain whether observed associations are causal or explained by unmeasured confounding or reverse causation.

**Methods:**

Using data from the Norwegian Mother, Father, and Child Cohort Study (MoBa) and Norwegian National Patient Registry, we investigated phenotypic associations between childhood LTCs, and depression and anxiety diagnoses in youth (<19 years), defined using ICD-10 diagnoses and self-rated measures. We then conducted two-sample Mendelian Randomization (MR) analyses using SNPs associated with childhood LTCs from existing genome-wide association studies (GWAS) as instrumental variables. Outcomes were: (i) diagnoses of major depressive disorder (MDD) and anxiety disorders or elevated symptoms in MoBa, and (ii) youth-onset MDD using summary statistics from a GWAS in iPSYCH2015 cohort.

**Results:**

Having any childhood LTC phenotype was associated with elevated youth MDD (OR = 1.48 [95% CIs 1.19, 1.85], p = 4.2×10^−4^) and anxiety disorder risk (OR = 1.44 [1.20, 1.73], p = 7.9×10^−5^). Observational and MR analyses in MoBa were consistent with a causal relationship between migraine and depression (IVW OR = 1.38 [1.19, 1.60], p_FDR_ = 1.8x10^−4^). MR analyses using iPSYCH2015 did not support a causal link between LTC genetic liabilities and youth-onset depression or in the reverse direction.

**Conclusions:**

Childhood LTCs are associated with depression and anxiety in youth, however, little evidence of causation between LTCs genetic liability and youth depression/anxiety was identified from MR analyses, except for migraine.

## Introduction

It is estimated that 20–40% of children worldwide have a long-term physical health condition (LTC) (Finning, Neochoriti Varvarrigou, Ford, Panagi, & Ukoumunne, 2022; Panagi, White, et al., 2022b; Panagi, Newlove-Delgado, et al., 2022a), and the prevalence of certain childhood LTCs, such as asthma, obesity, and diabetes, has increased in recent years (Abarca-Gómez et al., 2017; Dharmage, Perret, & Custovic, 2019; Royal College of Paediatrics and Child Health, 2020; Van Cleave, Gortmaker, & Perrin, 2010). It is well-established that children with LTCs are more likely to experience depression and anxiety than those without LTCs, particularly those with neurological illnesses such as epilepsy, migraine, and chronic fatigue syndrome (Finning et al., 2022; Glazebrook, Hollis, Heussler, Goodman, & Coates, 2003; Pinquart & Shen, 2011a, 2011b; Suryavanshi & Yang, 2016).

This association between LTCs and anxiety/depression has been attributed to many different mediators including social stigma (Bakula et al., 2019), poor self-esteem (Pinquart, 2013b), difficulties with peer relationships and bullying (Pinquart, 2017; Pittet, Berchtold, Akré, Michaud, & Surís, 2010), difficulties in the family environment (Pinquart, 2013a) (Qiu et al., 2021), and stress caused by the LTC itself (Compas, Jaser, Dunn, & Rodriguez, 2012). These are in addition to the potential physiological impacts of LTCs on the brain, particularly for neurological disorders such as epilepsy (Agrawal & Govender, 2011). Other exacerbating factors may include increased school absenteeism (Finning et al., 2022) and poorer academic performance/educational attainment (Hughes et al., 2021; Lum et al., 2017). However, observational studies also indicate that children with LTCs have similar levels of life satisfaction as their peers (Blackwell et al., 2019).

Residual confounding from unmeasured confounders remains a possibility in all observational studies, as well as pleiotropy (genetic overlap between LTCs and mental health conditions (Zhu et al., 2019)). Additionally, for some LTCs, such as migraine, the direction of association is unclear, that is, whether migraine causes an increased risk of anxiety/depression, or whether anxiety/depression increases the risk of migraine (Dyb, Stensland, & Zwart, 2015; Falla et al., 2022). Therefore, it is currently unclear whether LTCs have causal effects on youth anxiety and depression. Identifying causal mechanisms that underlie the development of youth anxiety and depression is important for developing effective prevention and early intervention strategies (Thapar, Eyre, Patel, & Brent, 2022).

Mendelian randomization (MR) is one method for inferring causation that has some analogies with a randomized controlled trial (Smith & Ebrahim, 2003). It uses randomly allocated (via meiosis) genetic variants, identified from genome-wide association studies (GWAS), as instrumental variables (IVs) for an exposure to test for a causal relationship between IVs (for the exposure) and outcome (Sanderson et al., 2022). When observational studies are supported by other designs, including MR, this triangulation improves the inference of causation between an exposure and outcome, given the assumptions of MR are met.

We aim to assess whether the relationship between LTCs and depression/ anxiety in youth is causal, by: (i) directly testing for longitudinal associations in a large cohort and (ii) using MR. We hypothesized that childhood LTCs, particularly neurological conditions such as migraine and epilepsy, would show a causal relationship with youth depression and anxiety.

## Methods

### Sample

We used data from the Norwegian Mother, Father, and Child Cohort Study (MoBa) (Magnus et al., 2016; Paltiel et al., 2014), a population-based pregnancy cohort study conducted by the Norwegian Institute of Public Health. Participants were recruited from Norway between 1999 and 2008. Women consented to participation in 41% of the pregnancies. The cohort includes ~114,500 children, 95,200 mothers, and 75,200 fathers. The current study is based on version 12 of the quality-assured data files released for research in January 2019. The establishment of MoBa and initial data collection was based on a license from the Norwegian Data Protection Agency and approval from The Regional Committees for Medical and Health Research Ethics. The MoBa cohort is regulated by the Norwegian Health Registry Act. The current study was approved by The Regional Committees for Medical and Health Research Ethics (2016/1702). All procedures contributing to this work comply with the ethical standards of the relevant national and institutional committees on human experimentation and with the Helsinki Declaration of 1975, as revised in 2008. Questionnaires were sent to mothers at 0.5, 1.5, 3, 5, 7, and 8 years and to mothers and children at 14 years. Blood samples were obtained from both parents during pregnancy and from mothers and children (umbilical cord) at birth (Paltiel et al., 2014). For MoBa genotyping information, including quality control, see Corfield et al. (2022).

### LTCs definition

To identify children with LTCs, we used definitions established in previous consensus (Finning et al., 2022; Mokkink et al., 2008; Panagi, et al., 2022a; Panagi et al., 2022b), whereby a disease or condition is considered to be a chronic/long-term condition in childhood if: (1) it occurs in children aged 0–18 years; (2) the diagnosis is based on medical scientific knowledge and can be established using reproducible and valid methods or instruments according to professional standards; (3) it is not (yet) curable and (4) it has been present for longer than 3 months or it will, very probably, last longer than 3 months, or it has occurred three times or more during the past year and will probably reoccur. Using this definition and the data available we included the following conditions: (i) arthritis, (ii) asthma, (iii) cerebral palsy, (iv) chronic fatigue syndrome, (v) coeliac disease, (vi) diabetes (type 1), (vii) epilepsy, (viii) migraine, and (ix) reduced hearing/hearing loss. In MoBa, all disorders were reported by the mother at age 14, except for cerebral palsy which was reported by mothers at age 5. We also included obesity, which was defined as BMI ≥95th percentile, calculated using the child’s reported height and weight at age 14. See Table 1 for *N.*

**Table 1.** Prevalence of LTCs

LTC	Total		Females	Males
	% with LTC	N with LTC	% with LTC	% with LTC
Asthma	12.0%	2097/17483	10.6%	13.3%
Migraine	8.7%	1520/17507	9.9%	7.5%
Obesity	5.1%	936/18525	5.0%	5.1%
Reduced hearing	2.3%	408/17501	2.2%	2.5%
Coeliac disease	1.9%	329/17493	2.3%	1.4%
Epilepsy	0.9%	153/17522	0.9%	0.8%
Diabetes	0.7%	126/17472	0.7%	0.8%
Chronic fatigue syndrome	0.3%	61/17506	0.5%	0.2%
Arthritis	0.3%	57/17523	0.4%	0.2%
Cerebral Palsy	0.2%	77/41233	0.2%	0.2%
*Any LTC specified above*	*28.4%*	*5039/17729*	*28.5%*	*28.1%*

### Mental health outcomes

Anxiety and depression were primarily defined in MoBa using the linked Norwegian Patient Registry, providing health records from MoBa participants from 2008 up to the date of 23 June 2021. We used ICD-10 codes of F32 (unipolar depressive episode) and F33 (recurrent depressive disorder), to define a major depressive disorder (MDD) diagnosis, and F40 (phobic anxiety disorders) and F41 (other anxiety disorders, including panic disorder and generalized anxiety disorder) to define an anxiety disorder. Since our focus is on youth depression and anxiety, outcomes were defined as a diagnosis before 19 years of age. Because MoBa has rolling recruitment (participants recruited between 1999 and 2008), not all participants reached 19 years of age by the time of administrative censoring. Therefore, descriptive frequencies of depression and anxiety disorders in youth were limited to children born before 2003 (minimum of 18.5 years of age at time of censoring, since exact birth dates were not available).

Mother- and self-reported depression and anxiety symptoms in children at age 14 were used as secondary outcomes. Self-reported depression symptoms were measured using the 13-item Short Mood and Feelings Questionnaire (SMFQ) (Angold, Costello, Messer, & Pickles, 1995) and the 5-item Screen for Child Anxiety Related Disorders (SCARED) (Birmaher et al., 1997; Birmaher et al., 1999), respectively. Total symptom scores were calculated for each scale (range: SMFQ = 0–26, SCARED = 0–10), and recommended cut-points were used to estimate probable depression (SMFQ ≥12) (Eyre et al., 2021), and anxiety disorder (SCARED ≥3) (Birmaher et al., 1999). Mother-report depression was measured using the shortened 6-item SMFQ (range 0–12).

### Statistical analysis

**Figure 1.**
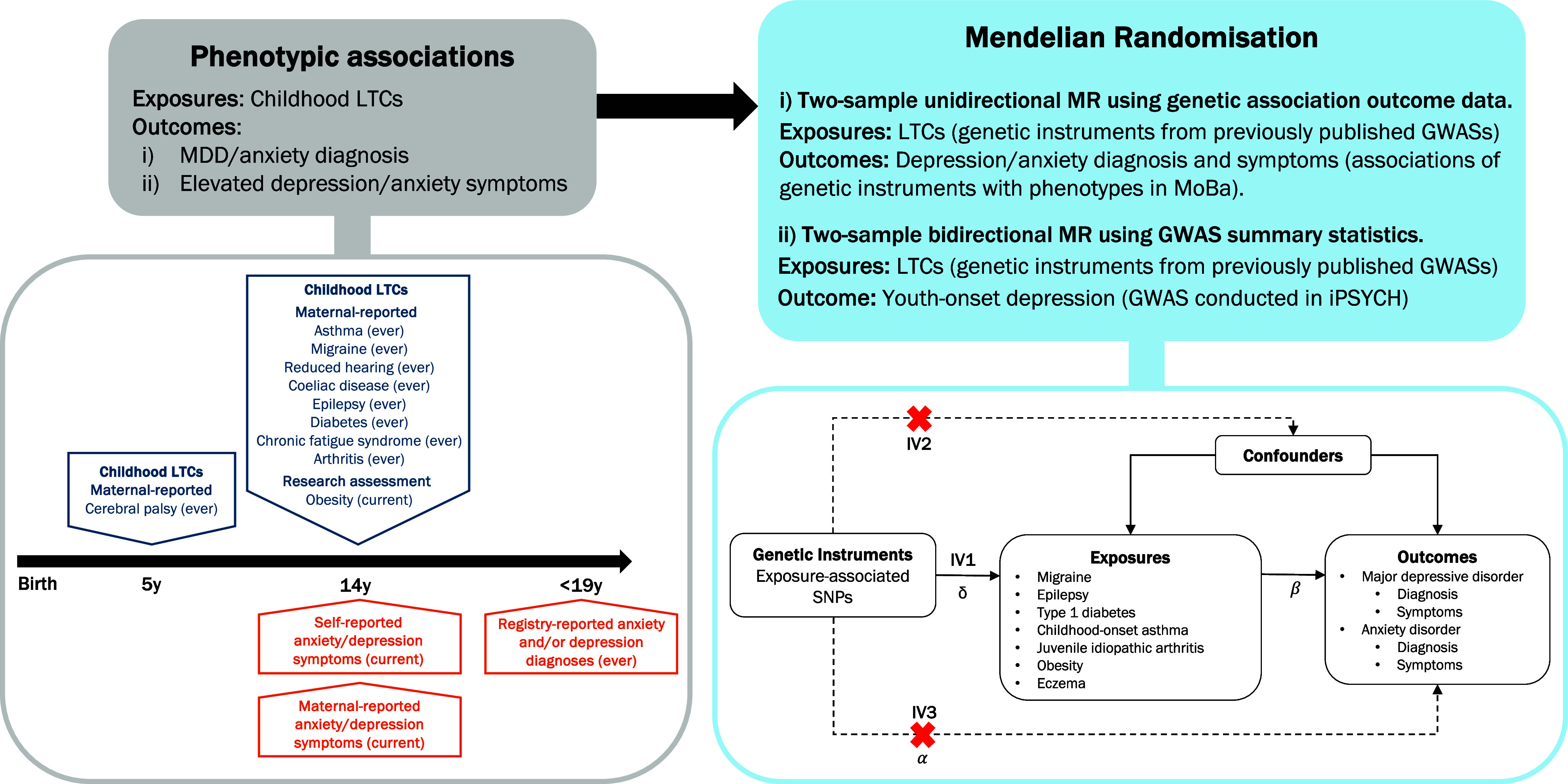
Investigating the association between LTC in childhood and youth anxiety/depression – study design. According to MR design, 𝛽 is the causal relationship of interest, where 𝛽 = 𝛼/δ. MR assumptions: IV1, Relevance = instruments are robustly associated with exposure; IV2, Independence = instruments are independent of any confounding variables; IV3, Exclusion restriction = instruments are independent of the outcome given exposure. LTC: long-term physical health conditions; SNPs: single nucleotide polymorphisms.

All analyses were conducted in R, version 4.2.1 (R Core Team, 2022). For an overview of the study design see Figure 1.

We determined the prevalence of each childhood LTC in the cohort and the combined prevalence of *any* childhood LTC at age 14. Chi-squared tests were used to establish whether LTC prevalence differed by sex. Associations between each separate LTCs were estimated using logistic regressions. We estimated the frequency of MDD or anxiety disorder diagnosis in youth, including only individuals who had reached the age of 18 at the time of administrative censoring.

#### Phenotypic associations

We tested for associations between childhood LTCs and mental health outcomes using logistic regressions. Birth year was included as a covariate in all analyses. Additionally, where there was a sex difference in the prevalence of childhood LTCs, sex was included as a covariate.

#### Two-sample Mendelian randomization

MR analyses are reported using STROBE-MR guidelines (Skrivankova et al., 2021).

##### Exposures

We identified genetic instruments from published childhood-onset specific GWAS for the following childhood LTCs: juvenile arthritis (Hinks et al., 2013), childhood-onset asthma (Ferreira et al., 2019), type 1 diabetes (Forgetta et al., 2020), childhood obesity (Bradfield et al., 2019), and genetic generalized epilepsy (International League Against Epilepsy Consortium on Complex Epilepsies et al., 2022). For migraine, where age-at-onset was not defined, the most recent and largest GWAS was used (Hautakangas et al., 2022). Descriptions of the samples used in each GWAS are presented in Supplementary Table S1. SNPs for use in MR analyses were identified as those associated with the phenotype at a genome-wide significant level (p < 5 × 10^−8^). Genetic instruments for eczema (Paternoster et al., 2015) were also included as an exposure of interest despite not being included in the phenotype analysis (since the relevant phenotypes were not available in MoBa). No suitable genetic instruments were available for cerebral palsy, chronic fatigue syndrome, coeliac disease, or hearing loss because of a lack of appropriate GWASs.

##### Outcomes

We used two approaches for defining outcomes in MR analyses. First, we used the same outcomes as in the phenotype analyses (MDD/anxiety diagnosis before 19 years and self-reported depression and anxiety symptoms at 14 years of age) in MoBa as outcomes for MR analyses. Here, we estimated the association between genetic instruments for each exposure with these phenotype outcomes in the MoBa genotyped cohort, including the first five ancestral principal components and genotyping batch number as covariates. Using this approach only one direction of association could be estimated (LTC exposures → MH outcomes).

Second, we performed two-sample bidirectional MR between childhood LTCs and youth-onset depression, using summary statistics from a GWAS of youth-onset MDD of 7,896 cases (diagnosed in a Danish psychiatric hospital, as an inpatient, outpatient, or in emergency settings, before the age of 19 years) and 23,590 controls (after controlling for relatedness and ancestry filtering) conducted in the Danish iPSYCH2015 cohort (Bybjerg-Grauholm et al., 2020; Pedersen et al., 2018). iPSYCH2015 is a large Danish registry-based genotyped case cohort established for the study of psychiatric disorders, see Supplementary material for more information. The threshold for identifying genetic instruments for the alternate direction MR (MDD → LTCs) was p < 5×10^−6^ and *F*-statistic >10 (although in practice no SNPs had *F*-statistic <20, indicating results are unlikely to suffer from weak instrument bias), leaving 15 SNPs as IVs.

For both approaches, we harmonized the outcome estimates with the exposure variants, so the effect estimates were expressed per effect allele increase. Where effect allele frequencies were not available in exposure/outcome data and harmonization was not possible because of being palindromic, SNPs were excluded from analyses. We used inverse-variance weighted (IVW) regression as the primary MR method though estimates were also generated using weighted median, weighted mode, MR-Egger (Bowden, Davey Smith, & Burgess, 2015) and MR-PRESSO (the MR pleiotropy residual sum and outlier, Verbanck, Chen, Neale, and Do (2018)) to assess horizontal pleiotropy and MR assumptions (Slob & Burgess, 2020). For details of MR methods see Supplementary material. A consistent effect across all methods provides evidence for a causal effect (Lawlor, Tilling, & Davey Smith, 2016). Cochran’s *Q* statistic was used to test for heterogeneity in instrument effects, whereby if *Q* > degrees of freedom, this provides evidence for heterogeneity and invalid instruments. Steiger tests of directionality (Hemani, Tilling, & Davey Smith, 2017) were also run to test directionality in the causal effect by examining the variance explained by IVs on exposures and outcomes.

MR analysis was carried out using the TwoSampleMR (Hemani et al., 2018) and MR-PRESSO (Verbanck, Chen, Neale, & Do, 2018) R packages.

We used the false discovery rate (FDR) to correct for multiple comparisons within phenotypic and within MR analysis, across exposures tested against each outcome (*n* = 10 for phenotypic analyses and *n* = 7 for MR). A *q*-value of 0.05 was used to define the FDR threshold.

### Sensitivity analysis

To assess temporal ordering, we tested associations between the same childhood LTCs and *subsequent* depression/anxiety diagnoses in MoBa, by restricting cases to those with a depression/anxiety diagnosis between the ages of 14 and 18 years (i.e. subsequent to mother-reporting of LTC), using the same method as in the main analysis. We also performed additional analyses testing for associations between childhood LTCs and total (continuous) self-reported depression and anxiety symptom scores and mother-reported depression symptom scores at age 14.

MR analyses using the largest GWAS for depression (Als et al., 2023) (excluding UK biobank) and anxiety (Purves et al., 2020) were also performed and reported in the Supplementary Material. These were not used in the primary analysis since they focus on depression and anxiety in adults, and evidence suggests that the genetic architecture of these disorders may differ across age-at-onset (Harder et al., 2022; Nguyen et al., 2023; Thapar & Riglin, 2020). Purves et al. (2020) anxiety disorder GWAS was conducted using data from the UK biobank study, from which cohort members were also included in some of the exposure GWAS for childhood LTCs (see Supplementary Table S1; migraine and childhood-onset asthma GWAS). Therefore, the type 1 error rate may be increased for these overlapping-sample MR sensitivity analyses.

## Results

### Descriptive information

Overall, 28% of children in the sample had at least one LTC reported at age 14. The numbers with and without each LTC are presented in Table 1. There was a male preponderance for asthma (p = 5.2×10^−8^), and a female preponderance for migraine (p = 3.0×10^−8^), coeliac disease (p = 1.1×10^−5^), and chronic fatigue syndrome (p = 8.9×10^−4^). Associations between the presence of each LTC with another are presented in Supplementary Table S2.

In the total cohort, 4.5% (716/15,958) had MDD, 4.9% (783/15,974) had an anxiety disorder, and 1.7% (262/15,827) had comorbid anxiety and depression diagnoses, with age-at-onset by 19 years as recorded in the linked healthcare registry data. Using questionnaire data at age 14, 26.8% (5,785/21,549) reported elevated anxiety symptoms, and 21.5% (4554/21,229) reported elevated depression symptoms. All anxiety and depression outcomes were more common in females (Supplementary Table S3).

### Phenotypic associations between childhood LTCs and youth anxiety/depression

**Figure 2.**
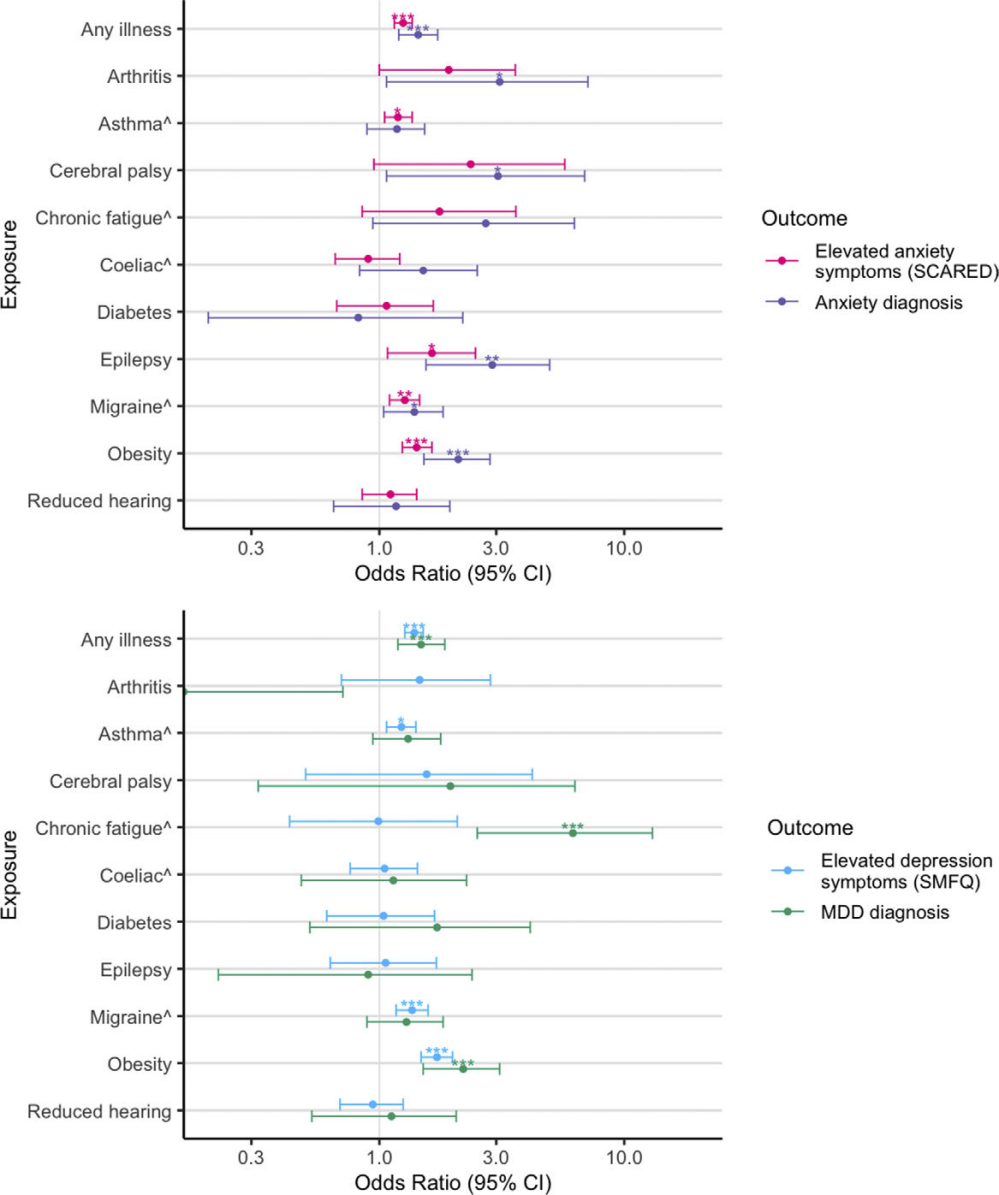
Phenotypic associations between LTCs at age 14 and anxiety diagnosis (top, purple) and symptoms (top, pink) measured by the SCARED questionnaire, and major depressive disorder diagnosis (bottom, green) and depression symptoms (bottom, blue) measured by the SMFQ questionnaire. LTCs with ^ indicate sex was included as a covariate in the analysis. Cerebral palsy was reported by mothers when children were aged 5 years. *p < 0.05; **p < 0.01; ***p < 0.001 (FDR adjusted p-values across 10 LTCs). Test statistics are presented in Supplementary Table S4.

Having any LTC was associated with anxiety (OR = 1.44 [1.20, 1.73], p = 7.9×10^−5^) and depression diagnoses in youth (OR = 1.48 [1.19, 1.85], p = 4.2×10^−4^), as well as with elevated anxiety (OR = 1.25 [1.15, 1.36], p = 1.4×10^−7^) and depression symptoms (OR = 1.39 [1.27, 1.51], p = 7.5×10^−13^) (Figure 2). Specifically, the following childhood LTCs were associated with an increased risk of anxiety diagnosis in youth: arthritis, cerebral palsy, epilepsy, migraine, and obesity. Similarly, epilepsy, migraine, obesity, and additionally asthma, were also associated with elevated self-reported anxiety symptoms at age 14. Increased risk of MDD diagnosis was found for chronic fatigue syndrome and obesity. Asthma, migraine, and obesity were associated with elevated self-reported depressive symptoms.

### MR analysis

**Figure 3.**
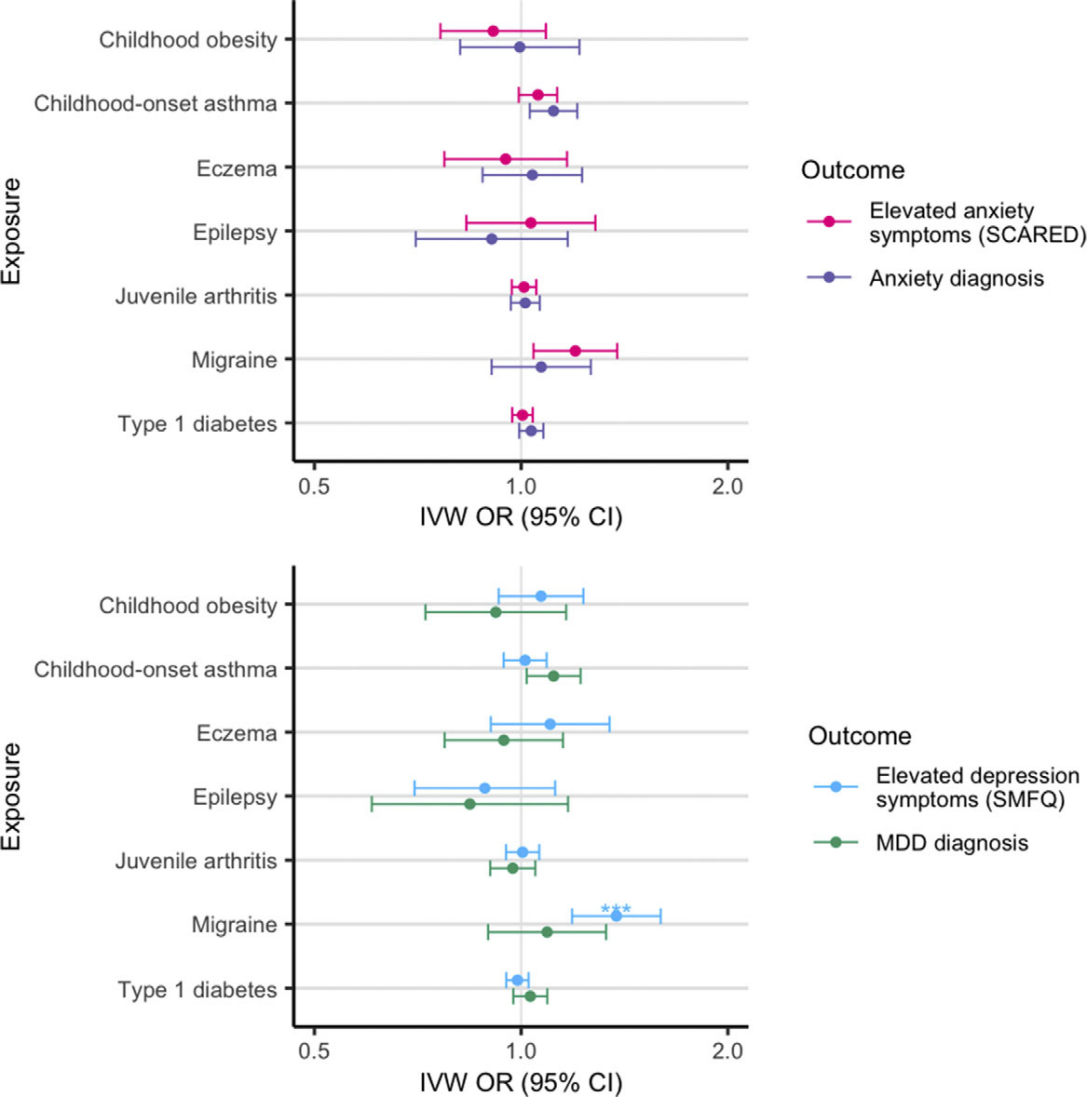
MR analyses testing for evidence of causality between childhood LTCs and anxiety diagnosis (top, purple) and symptoms (top, pink) measured by the SCARED questionnaire, and major depressive disorder diagnosis (bottom, green) and depression symptoms (bottom, blue) measured using SMFQ in the MoBa dataset. IVW OR = Inverse variance weighted MR odds ratio estimate. *** FDR corrected *p* < 0.001. See Supplementary Table S5 for all test statistics.

In MR analyses using MoBa data for outcomes, there was some evidence of causality between genetic liability to childhood-onset asthma and risk of MDD (IVW OR = 1.11 [1.02, 1.21], p = 0.02, p_FDR_ = 0.13) and anxiety disorder diagnoses (IVW OR = 1.11 [1.03, 1.21], p = 0.008, p_FDR_ = 0.05). There was little evidence of causal links between genetic liability to other LTCs and MDD/anxiety disorders (Figure 3, Supplementary Table S5). We found stronger causal evidence of genetic liability to migraine and our secondary outcome measures: elevated self-reported depression symptoms (IVW OR = 1.38 [1.19, 1.60], p = 2.6×10^−5^, p_FDR_ = 1.8×10^−4^, see Supplementary Figure S1 for MR scatter plot) and elevated self-reported anxiety symptoms (IVW OR = 1.20 [1.04, 1.38], p = 0.01, p_FDR_ = 0.08) at age 14.

We found limited evidence of horizontal pleiotropy using MR Egger (Supplementary Table S6), and the estimates of MR Egger, weighted median, and mode MR estimates agreed with IVW for these results (Supplementary Table S5). Results from MR-PRESSO global tests for estimates where we found evidence of potential causal links (childhood-onset asthma and MDD/anxiety, and migraine and anxiety/depression symptoms) did not indicate outliers (Supplementary Table S7). There was limited evidence for heterogeneity in IV effects for the same estimates, see Supplementary Table S8. *F* statistics for all exposures are presented in Supplementary Table S9. Steiger directionality tests indicated the correct causal direction for MR analyses where we found evidence of potential causal links (Supplementary Table S10).

Bidirectional MR analysis using GWAS of youth-onset depression (in the iPSYCH2015 cohort) did not indicate strong evidence for causality between genetic liability to any childhood LTC and youth-onset depression (Table 2), nor in the alternate direction (Supplementary Table S11). MR Egger intercepts indicated limited evidence of horizontal pleiotropy for exposures/outcomes (Supplementary Table S12). Estimates from sensitivity analysis agreed with the main IVW analysis. Details of *F* statistics for all instruments are presented in Supplementary Table S9 and results from heterogeneity tests in Supplementary Table S13.

**Table 2.** Results from Mendelian Randomisation (MR) analysis of childhood LTCs and youth-onset depression using iPSYCH2015 outcome data

Exposure	Outcome	MR method	N SNPs	OR	95% CI		P-value	P_FDR_
					Lower	Upper		
**Childhood obesity**	**Youth-onset depression**	**Inverse variance weighted**	**8**	**0.99**	**0.9**	**1.09**	**0.87**	**0.99**
		MR Egger	8	0.89	0.57	1.40	0.64	
		Weighted median	8	0.98	0.87	1.12	0.80	
		Weighted mode	8	0.92	0.75	1.11	0.41	
**Childhood-onset asthma**	**Youth-onset depression**	**Inverse variance weighted**	**86**	**0.98**	**0.94**	**1.02**	**0.33**	**0.74**
		MR Egger	86	0.95	0.86	1.05	0.31	
		Weighted median	86	0.99	0.93	1.06	0.86	
		Weighted mode	86	1.00	0.92	1.10	0.95	
**Eczema**	**Youth-onset depression**	**Inverse variance weighted**	**10**	**0.99**	**0.88**	**1.12**	**0.88**	**0.99**
		MR Egger	10	1.20	0.73	1.98	0.48	
		Weighted median	10	0.96	0.84	1.10	0.60	
		Weighted mode	10	0.96	0.78	1.17	0.67	
**Genetic generalized epilepsy**	**Youth-onset depression**	**Inverse variance weighted**	**19**	**1.12**	**0.91**	**1.37**	**0.28**	**0.74**
		MR Egger	19	0.79	0.25	2.54	0.70	
		Weighted median	19	1.07	0.85	1.35	0.54	
		Weighted mode	19	1.07	0.74	1.54	0.73	
**Juvenile idiopathic arthritis**	**Youth-onset depression**	**Inverse variance weighted**	**4**	**1.01**	**0.98**	**1.05**	**0.43**	**0.74**
		MR Egger	4	1.09	0.99	1.20	0.22	
		Weighted median	4	1.02	0.98	1.06	0.30	
		Weighted mode	4	1.03	0.99	1.07	0.27	
**Migraine**	**Youth-onset depression**	**Inverse variance weighted**	**96**	**1**	**0.91**	**1.1**	**0.99**	**0.99**
		MR Egger	96	0.79	0.61	1.01	0.07	
		Weighted median	96	0.96	0.83	1.11	0.56	
		Weighted mode	96	0.96	0.78	1.19	0.72	
**Type 1 diabetes**	**Youth-onset depression**	**Inverse variance weighted**	**40**	**0.99**	**0.97**	**1.01**	**0.28**	**0.74**
		MR Egger	40	0.97	0.94	1.00	0.07	
		Weighted median	40	0.98	0.96	1.01	0.19	
		Weighted mode	40	0.99	0.96	1.01	0.26	

### Sensitivity analysis

***Phenotypic associations***. Associations between childhood LTCs and *subsequent* anxiety and MDD (specifically, diagnosed after the LTC, between ages 14 and 18 years) showed a similar pattern of results as the main phenotype analysis, with evidence for associations between any childhood LTC and both anxiety (OR = 1.37 [1.09, 1.71], *p* = 0.006) and depression (OR = 1.55 [1.21, 1.97], *p* = 4.7×10^−4^) diagnoses. For associations of individual LTCs see Supplementary Table S14.

There were similar associations of childhood LTC with total (continuous) depression and anxiety symptom scores (Supplementary Table S15), with stronger evidence of an association between cerebral palsy and elevated self-reported anxiety symptom scores at age 14 (*b* = 1.37 ± 0.43, *p* = 0.001), as well as between arthritis and elevated self-reported depressive symptom scores at age 14 (*b* = 2.15 ± 0.95, *p* = 0.02).

There were associations between asthma, chronic fatigue syndrome, diabetes, epilepsy migraine, obesity, and reduced hearing and mother-reported depressive symptom scores at age 14 (see Supplementary Table S16), although mother- and self-reported SMFQ scores at age 14 were only modestly correlated (Spearman’s *r* = 0.36, p < 2.2x10^−6^).

***MR using adult anxiety and depression GWAS***. Two-sample MR testing for causality between childhood LTCs and depression/anxiety using the largest available GWASs from adult samples (Als et al., 2023; Purves et al., 2020) indicated some evidence that genetic liability to migraine is causally linked with depression, and genetic liability to type 1 diabetes and decreased risk of an anxiety disorder (Supplementary Table S17); however, these estimates did not survive FDR correction. There was also limited evidence of pleiotropy for these estimates (Supplementary Table S18).

## Discussion

With the prevalence of youth anxiety and depression increasing globally (Thapar et al., 2022, it is vital to identify potentially causal risk factors. Rates of LTCs have also risen across a similar time period (Van Cleave et al., 2010) indicating a potential causal factor, although social changes are also important (Collishaw, 2015). In this study, we investigated the association between LTCs and anxiety/depression in young people using phenotypic and genomic data from a large cohort with linked medical registry data. Consistent with previous literature, we observed phenotypic associations between several childhood LTCs and depression and anxiety disorders and symptoms, with neurological conditions such as epilepsy and migraine, as well as obesity, demonstrating the highest risk. We then used MR as a test of causality. However, our MR findings did not indicate causal relationships between most LTCs and youth anxiety and depression, with the exception of migraine.

Our observed phenotypic associations between childhood LTCs and depression/anxiety are comparable to estimates from previous literature, demonstrating associations between a range of LTCs and clinically diagnosed anxiety/depression as well as elevated anxiety and depression symptoms in youth. Overall, we saw a 48% increase in MDD and a 44% increase in anxiety disorder diagnosis in young people with any LTC compared with those without. In line with previous evidence, we observed associations between arthritis (Fair, Rodriguez, Knight, & Rubinstein, 2019), asthma (Dudeney, Sharpe, Jaffe, Jones, & Hunt, 2017), chronic fatigue syndrome (Loades et al., 2021), epilepsy (Ekinci, Titus, Rodopman, Berkem, & Trevathan, 2009), migraine (Falla et al., 2022), and obesity (Lindberg, Hagman, Danielsson, Marcus, & Persson, 2020) with elevated anxiety. For depression, we observed associations with asthma (Chen et al., 2014), chronic fatigue syndrome (Bould, Collin, Lewis, Rimes, & Crawley, 2013), migraine (Falla et al., 2022), and obesity (Lindberg et al., 2020). Unlike previous studies we did not see strong evidence of worse mental health in young people with type 1 diabetes (Buchberger et al., 2016; Liu et al., 2022), reduced hearing/hearing loss (Stevenson, Kreppner, Pimperton, Worsfold, & Kennedy, 2015), or coeliac disease (Coburn, Puppa, & Blanchard, 2019), although previous evidence for coeliac disease is varied.

However, MR analyses did not support a causal interpretation of the observational associations between the majority of childhood LTCs and youth anxiety/depression, with the only consistent association seen in both observational and MR estimates being for migraine and depression symptoms as well as weaker evidence for anxiety symptoms. There was also some evidence that genetic liability to child-onset asthma could have causal effects on depression and anxiety disorders using MR analysis in MoBa data, however, these associations were not present in the observational analysis.

There are several potential explanations for our findings. First, the observed phenotypic associations between childhood LTCs and anxiety/depression are not causal. Previous literature indicates a number of potential confounders, including shared risk factors for LTCs and anxiety/depression (e.g. pre-/peri-natal factors (Fitzallen, Sagar, Taylor, & Bora, 2021, Heikkila et al., 2021), family adversities (Davies et al., 2016; Kaasboll, Skokauskas, Lydersen, & Sund, 2021; Kinnunen et al., 2021; Sieh, Meijer, Oort, Visser-Meily, & Van der Leij, 2010; Vejrup, Hillesund, Agnihotri, Helle, & Øverby, 2023), lifestyle factors (Liu et al., 2022; Sundell & Angelhoff, 2021), and poverty (Lai et al., 2019, Najman et al., 2010, Royal College of Paediatrics and Child Health, 2020, Thapar et al., 2022). To our knowledge, a thorough investigation of the effect of these confounding factors on observed associations has not yet been undertaken. However, it has been shown that residual confounding remains a problem in all observational designs (Fewell, Davey Smith, & Sterne, 2007; Thapar & Rutter, 2019). For this reason, it is important to use alternate designs to infer causality. Additionally, there is the possibility of other shared genetic risk factors (independent of genetic instruments used in the current study which did not show evidence of horizontal pleiotropy) contributing both to LTCs and depression/anxiety, since previous evidence suggests genetic overlap for some LTCs (Zhu et al., 2019).

Longitudinal and MR estimates did not suggest evidence for reverse causation (that genetic liability to anxiety/depression could lead to childhood LTCs), nor does the normal temporal pattern of condition emergence support this, with many childhood LTCs onsetting in young childhood (such as cerebral palsy and asthma), whereas the typical age of onset for depression and many types of anxiety is during adolescence (McGrath et al., 2023, Thapar et al., 2022). For LTCs with a more variable age-of-onset, such as migraine, type 1 diabetes, and chronic fatigue syndrome, we were able to use bidirectional MR analyses to test for the presence of reverse causation, where we observed little causal evidence in the direction of youth-onset depression to LTC.

Our MR findings suggested that LTCs, excluding migraine, do not appear to be a causal risk factor for youth anxiety or depression. This is important as it suggests that emotional disorders are not unavoidable in children and adolescents diagnosed with an LTC, since mechanisms other than a causal relationship may explain observed associations (e.g. poverty, early lifestyle factors, and family environment). It also suggests that LTCs are unlikely to explain the recent increase in rates of anxiety and depression in young people, although the causal relationship should be assessed using other designs as there are limitations to MR.

An exception to our findings was the potentially causal relationship between migraine and increased risk of depression/anxiety at age 14, with MR estimates in agreement with observational estimates. These results were supplemented with our MR analysis using the largest adult depression GWAS as an outcome (Als et al., 2023), which also indicated weaker evidence of a causal relationship between genetic liability to migraine and lifelong depression risk. However, we did not find evidence consistent with a causal effect of genetic liability to migraine on a clinical MDD diagnosis in analyses using both MoBa and iPSYCH2015 data, potentially indicating different effects of migraine on depression symptoms versus a clinical diagnosis in young people. There is a large body of research on the links between migraine and depression, suggesting a variety of biopsychosocial mechanisms that might contribute to the association. These have included shared genetic risk, brain neurobiology, and environmental factors such as stress (Baksa, Gonda, & Juhasz, 2017; Wachowska et al., 2023).

These results should be considered in light of several methodological points. The first is the potential of low statistical power/measurement error for MR analyses because of the limited availability of robust genetic instruments for some exposures, meaning that the lack of an observed causal effect between exposures and outcomes does not rule out the presence of a causal relationship. We recommend that other designs are used to triangulate evidence from this study including traditional genetic studies such as twin designs. We were stringent about our selection of genetic instruments in that we prioritized GWAS with a child/adolescent focus, which could be considered a limitation since larger (non-age-specific) GWAS may provide more genetic instruments. However, we opted for this approach because the genetic architecture of LTCs with childhood-onset are not the same as typically adult-onsetting LTCs, for example childhood versus adult-onset asthma (Ferreira et al., 2019) and type 1 versus type 2 diabetes (Aylward, Chiou, Okino, Kadakia, & Gaulton, 2018), and similarly is the case for youth versus adult-onset depression (Nguyen et al., 2023).

A further limitation is that we used mother-reporting of offspring LTCs because of the limited availability of LTC diagnostic data. This meant that we were not able to capture all childhood LTCs, only those included in the questionnaires. Further, we cannot be sure that mother-reporting was completely accurate. However, evidence from developmental disorders and other child health outcomes indicate that parent reporting can generally be considered reliable (DiLalla, Trask, Casher, & Long, 2020; Miller, Perkins, Dai, & Fein, 2017; Orzan et al., 2021; Shaikh, Nettiksimmons, Bell, Tancredi, & Romano, 2012), and prevalence rates of LTCs in this study are congruent with published rates. Further, we were unable to stratify offspring LTC by illness severity which may be an important contributor to mental health outcomes (Brady, Deighton, & Stansfeld, 2021). Despite these limitations, we observed phenotypic associations.

Although these results do not provide support for a causal link between most childhood LTCs and anxiety/depression in youth, it has been consistently shown, across international cohorts, that young people with LTCs have higher rates of mental disorders (Finning et al., 2022; Pinquart & Shen, 2011b; Suryavanshi & Yang, 2016). Therefore, this should be considered in the provision of services and support for these children, especially in light of the increasing rates of both physical and mental health conditions in young people globally (Abarca-Gómez et al., 2017; Dharmage et al., 2019; Royal College of Paediatrics and Child Health, 2020; Thapar et al., 2022; Van Cleave et al., 2010).

## Conclusion

Evidence from this study substantiates previous evidence that children and young people with LTCs experience higher rates of depression and anxiety than their physically healthy counterparts. However, using MR analysis, we saw limited evidence to support causal relationships between common childhood LTCs and anxiety and depression, highlighting the need for further investigations to understand the observed associations between childhood LTCs and youth mental health.

## Supporting information

Shakeshaft et al. supplementary material Shakeshaft et al. supplementary material

## Data Availability

Data from the Norwegian Mother, Father, and Child Cohort Study and the Medical Birth Registry of Norway used in this study are managed by the national health register holders in Norway (Norwegian Institute of Public Health) and can be made available to researchers, provided approval from the Regional Committees for Medical and Health Research Ethics (REC), compliance with the EU General Data Protection Regulation (GDPR) and approval from the data owners. The consent given by the participants does not open for storage of data on an individual level in repositories or journals. Researchers who want access to data sets for replication should apply through helsedata.no. Access to data sets requires approval from The Regional Committee for Medical and Health Research Ethics in Norway and an agreement with MoBa.

## Supplementary material

To view supplementary material for this article, please visit http://doi.org/10.1017/S0033291724003271.
