# Supplementary material for: Long-term physical health conditions and youth anxiety and depression: Is there a causal link?
Source: Psychol Med. 2025 Feb 4;55:e7. doi: 10.1017/S0033291724003271 (PMC11968126; doi:10.1017/S0033291724003271)
Supplement: Shakeshaft et al. supplementary material [file S0033291724003271sup001.docx]

# Supplementary Material

## Supplementary Text

**IPSYCH Sample and GWAS**

We used data from the iPSYCH2015 case-cohort study sample (Bybjerg-Grauholm *et al.*, 2020), an update of the iPSYCH2012 case-cohort (Pedersen *et al.*, 2018). The study includes 141,265 individuals selected from all 1,657,449 singleton births that occurred in Denmark between 1^st^ May 1981, and 31^st^ Dec 2008, who were alive and residing in Denmark on their first birthday and had a mother registered in the Danish Civil Registration System (Pedersen, 2011). This study includes cases as individuals with one of more of the following psychiatric illnesses registered in the Danish Psychiatric Central Research Register (Mors *et al.*, 2011) (established in 1970) no later than 31^st^ Dec 2015: schizophrenia spectrum disorder (ICD-10 F20–F29; n=16,008), bipolar disorder (ICD-10 F30–F31; n=3,819), major depressive disorder (ICD-10 F32–F33 and ICD-8 296.09, 296.29, 298.09, and 300.49; n=37,555), autism spectrum disorder (ICD-10 F84; n=24,975), or ADHD (ICD-10 F90; n=29,668). The cohort comprised 50,615 individuals drawn from the source population at random. Where available a dried blood spot was obtained from the Danish Neonatal Screening Biobank (Hollegaard *et al.*, 2011). The use of this data is according the guidelines provided by the Danish Scientific Ethics Committee, the Danish Health Data Authority, the Danish data protection agency and the Danish Neonatal Screening Biobank Steering Committee. For individuals in the case-cohort sample, all available biobanked blood samples underwent DNA extraction, genotyping, and quality-control filtering as part of the iPSYCH2012 and iPSYCH2015 studies, as described below. Individuals without available samples for genotyping or with samples that did not pass quality control were excluded from further study.

For this study, we identified youth-onset depression cases in the iPSYCH cohort through Danish Psychiatric Central Research Register and Danish National Hospital Register. The ICD-10 codes used to identify cases were F23-33. Only individuals who received a diagnosis of depression prior to their 19^th^ birthday were included as cases. We excluded those we received a diagnosis of mania (F30), bipolar disorder (F31), or a psychotic disorder (F2) before or on the date of their first unipolar depression diagnosis. Controls were from the iPSYCH population representative subcohort who had turned 19 years old by 31^st^ Dec 2016.

Genetic data from iPSYCH2012 and iPSYCH2015 were imputed using the RICOPILI imputation pipeline (Lam *et al.*, 2020), and then combined into a single dataset. We restricted the analysis to SNPs that passed RICOPILI quality controls for both waves, resulting in a total of 8,785,478 SNPs for the GWAS. The analysis was restricted to a group of individuals with European ancestry, which were identified by calculating a robust Mahalanobis distance based on the first 20 PCs and restricting to a log-distance below 4.5 (Privé *et al.*, 2020). We filtered for relatedness by removing individuals (the second one in each pair) with a KING-relatedness above 0.088. The final number of individuals used for the GWAS was 7,896 MDD cases and 23,590 controls. The logistic regression GWAS was performed using the bigsnpr package (Privé *et al.*, 2018) for R. We used 20 PCs, sex, age and imputation wave as covariates for the analyses.

Using linkage disequilibrium score regression (Bulik-Sullivan *et al.*, 2015)), we note a SNP-heritability of 0.10, SE=0.017 for youth depression using these summary statistics.

**Mendelian randomisation methods**

We used inverse-variance weighted (IVW) regression as the primary MR method though estimates were also generated using weighted median, weighted mode, MR-Egger (Bowden *et al.*, 2015) and MR-PRESSO (Verbanck *et al.* (2018)) to assess horizontal pleiotropy and MR assumptions (Slob and Burgess, 2020). Weighted median and weighted mode methods are consensus methods, unbiased by invalid IVs and robust to outliers. MR-Egger, contains an intercept term which represents the average pleiotropic effect across all IVs, and gives unbiased estimates even in the presence of horizontally pleiotropy, conditional on the assumption that the pleotropic effects are distributed independently of instrument strength (instrument strength independent of direct effect; InSIDE). MR-PRESSO is an outlier-robust method consisting of three parts, (i) the global test which detects horizontal pleiotropy, (ii) the outlier corrected causal estimate which corrects for the detected horizontal pleiotropy and (iii) the distortion test which estimates if the causal estimate is significantly different (at p<0.05) after adjustment for outliers.

## Supplementary Tables

**Table S1** – GWAS studies of childhood LTCs used to identify genetic instruments for Mendelian Randomisation analysis.

| **Disease** | **Reference** | **Sample size (Case/control)** | **Number of IVs** | **Sample details & phenotype definitions** | **Note** |
| --- | --- | --- | --- | --- | --- |
| Type 1 Diabetes | (Forgetta *et al.*, 2020) | 9,266/  15,574 | 40 | 6 type 1 diabetes cohorts (some specify diagnosis <18y, others diagnosis <~31-35y). | Age for eligibility criteria unclear for some cohorts. |
| Migraine | (Hautakangas *et al.*, 2022) | 102,084/  771,257 | 96 | Self-reported migraine. Genome-wide meta-analysis on migraine included five study collections: IHGC2016, 23andMe, UKBB, GeneRISK and HUNT All-in Headache. | No suitable GWAS of childhood-onset migraine.  PheWAS of lead variants do no indicate pleiotropy with depression. |
| Childhood-onset asthma | (Ferreira *et al.*, 2019) | 13,962/  300,671 | 86 | Diagnosed with asthma as a child or teenager (<=19y) in UKBB. | Childhood and adult-onset asthma have genetic correlation of 0.67. |
| Eczema (atopic dermatitis) | (Paternoster *et al.*, 2015) | 10,788/  30,047 | 10 | Meta-analysis of eczema/atopic dermatitis from 20 cohorts. Mixture of self-report (5 studies [N=3023]) and Dr diagnosed (15 studies [N=7765]). 16 studies specify childhood-onset (N=7075), 4 studies onset at any age (N=3713). | MoBa contributes 206 cases and 413 controls with Dr diagnosed eczema by age 3 |
| Juvenile idiopathic arthritis | (Hinks *et al.*, 2013) | 2,816/  13,056 | 4 | UK, US and German cohorts of oligoarticular or rheumatoid factor-negative polyarticular, all paediatric cases. |  |
| Childhood obesity | (Bradfield *et al.*, 2019) | 8,613/  12,696 | 8 | From Early Growth Genetics Consortium. BMI >= 95th percentile at any point in childhood and controls BMI <= 50th percentile consistently throughout childhood for all available measures. | MoBa used only in replication sample. Only EUR results used. |
| Genetic generalised epilepsy (GGE) | (International League Against Epilepsy Consortium on Complex Epilepsies *et al.*, 2022) | 6,952/  42,436 | 19 | International League Against Epilepsy consortium. Meta-analysis of 5 epilepsy cohorts. GGE includes CAE, JME, JAE and GTCS only. | GGE not specifically childhood but younger average age of onset than overall epilepsy. |
| *BMI: body mass index; CAE: childhood absence epilepsy; GGE: genetic generalised epilepsy; GTCS only: epilepsy with generalised tonic clonic seizures only; GWAS: genome-wide association study; JAE: juvenile absence epilepsy; JME: juvenile myoclonic epilepsy; MoBa: Norwegian mother father and child birth cohort study; pheWAS: phenome-wide association study; UKBB: UK biobank study.* | | | | | |

**Table S2** – Odds ratios from logistic regression models testing for association between childhood long-term physical health condition.

|  | Asthma | Migraine | Obesity | Reduced hearing | Coeliac disease | Epilepsy | Diabetes | Chronic fatigue syndrome | Arthritis | Cerebral palsy |
| --- | --- | --- | --- | --- | --- | --- | --- | --- | --- | --- |
| Asthma |  | **1.60*** | 1.23 | **1.66*** | **1.39*** | 1.39 | 1.14 | 1.69 | 0.56 | 0.93 |
| Migraine |  |  | 1.04 | 1.32 | 1.22 | 1.59 | **0.17*** | **2.15*** | 1.01 | - |
| Obesity |  |  |  | **1.75*** | 0.47 | **2.35*** | 1.27 | 2.55 | 1.86 | 1.19 |
| Reduced hearing |  |  |  |  | 1.32 | 1.13 | 0.25 | 1.50 | 0.75 | 0.13 |
| Coeliac disease |  |  |  |  |  | 1.04 | **12.39*** | **3.82*** | 1.93 | <0.01 |
| Epilepsy |  |  |  |  |  |  | 0.90 | **8.49*** | 2.06 | **15.50*** |
| Diabetes |  |  |  |  |  |  |  | <0.01 | 2.47 | <0.01 |
| Chronic fatigue syndrome |  |  |  |  |  |  |  |  | <0.01 | <0.01 |
| Arthritis |  |  |  |  |  |  |  |  |  | <0.01 |
| Cerebral Palsy |  |  |  |  |  |  |  |  |  |  |
| **indicates p<0.05* | | | | | | | | | | |

**Table S3** – Prevalence of depression and anxiety outcomes, in total cohort, as well as stratified by sex.

| **Outcome** | **Total** | | **Males** | | **Females** | | |
| --- | --- | --- | --- | --- | --- | --- | --- |
|  | **N** | **%** | **N** | **%** | **N** | **%** |  |
| **Anxiety diagnosis <19y** | 783/15974 | 4.9% | 78/2504 | 3.1% | 157/2413 | 6.5% |  |
| **Elevated anxiety symptoms** | 5785/21549 | 26.8% | 1082/9420 | 11.5% | 4334/10914 | 39.7% |  |
| **MDD diagnosis <19y** | 716/15958 | 4.5% | 64/2505 | 2.6% | 129/2405 | 5.4% |  |
| **Elevated depression symptoms** | 4554/21229 | 21.5% | 896/9328 | 9.6% | 3359/10701 | 31.4% |  |

**Table S4 –** Results of logistic regressions between LTCs and mental health outcomes in MoBa. ^indicates where sex was included as a covariate in the model.

| LTC | Outcome | N | OR | LCI | UCI | Beta | SE | P | P_FDR_ |
| --- | --- | --- | --- | --- | --- | --- | --- | --- | --- |
| Any LTC | Anxiety diagnosis | 17679 | 1.44 | 1.2 | 1.73 | 0.37 | 0.09 | **7.9x10^-5^** | *-* |
|  | MDD diagnosis | 17680 | 1.48 | 1.19 | 1.85 | 0.4 | 0.11 | **4.2x10^-4^** | *-* |
|  | Elevated anxiety symptoms (SCARED) | 13539 | 1.25 | 1.15 | 1.36 | 0.22 | 0.04 | **1.4x10^-7^** | *-* |
|  | Elevated depression symptoms (SMFQ) | 13314 | 1.39 | 1.27 | 1.51 | 0.33 | 0.05 | **7.5x10^-13^** | *-* |
| Arthritis | Anxiety diagnosis | 17474 | 3.1 | 1.07 | 7.11 | 1.13 | 0.47 | **0.02** | **0.04** |
|  | MDD diagnosis | 17475 | 0 | 0 | 0.71 | -12.69 | 312.75 | 0.97 | 0.97 |
|  | Elevated anxiety symptoms (SCARED) | 13444 | 1.92 | 1 | 3.59 | 0.65 | 0.32 | 0.04 | 0.09 |
|  | Elevated depression symptoms (SMFQ) | 13222 | 1.46 | 0.7 | 2.84 | 0.38 | 0.35 | 0.28 | 0.70 |
| Asthma^ | Anxiety diagnosis | 16655 | 1.18 | 0.89 | 1.53 | 0.17 | 0.14 | 0.23 | 0.28 |
|  | MDD diagnosis | 16655 | 1.31 | 0.94 | 1.78 | 0.27 | 0.16 | 0.10 | 0.34 |
|  | Elevated anxiety symptoms (SCARED) | 12880 | 1.19 | 1.05 | 1.36 | 0.18 | 0.07 | **0.01** | **0.02** |
|  | Elevated depression symptoms (SMFQ) | 12665 | 1.23 | 1.07 | 1.41 | 0.21 | 0.07 | **3.2x10^-3^** | **0.01** |
| Cerebral palsy | Anxiety diagnosis | 41196 | 3.05 | 1.07 | 6.89 | 1.12 | 0.47 | **0.02** | **0.04** |
|  | MDD diagnosis | 41196 | 1.95 | 0.32 | 6.29 | 0.67 | 0.72 | 0.36 | 0.59 |
|  | Elevated anxiety symptoms (SCARED) | 13485 | 2.36 | 0.95 | 5.72 | 0.86 | 0.45 | 0.06 | 0.09 |
|  | Elevated depression symptoms (SMFQ) | 13343 | 1.56 | 0.5 | 4.21 | 0.44 | 0.53 | 0.40 | 0.81 |
| Chronic fatigue^ | Anxiety diagnosis | 16679 | 2.72 | 0.94 | 6.26 | 1 | 0.47 | 0.03 | 0.06 |
|  | MDD diagnosis | 16679 | 6.17 | 2.51 | 13.03 | 1.82 | 0.41 | **1.1x10^-5^** | **8.3x10^-5^** |
|  | Elevated anxiety symptoms (SCARED) | 12899 | 1.76 | 0.85 | 3.61 | 0.57 | 0.36 | 0.12 | 0.17 |
|  | Elevated depression symptoms (SMFQ) | 12682 | 0.99 | 0.43 | 2.08 | -0.01 | 0.4 | 0.98 | 0.98 |
| Coeliac^ | Anxiety diagnosis | 16665 | 1.51 | 0.83 | 2.51 | 0.41 | 0.28 | 0.14 | 0.20 |
|  | MDD diagnosis | 16665 | 1.14 | 0.48 | 2.27 | 0.13 | 0.39 | 0.73 | 0.94 |
|  | Elevated anxiety symptoms (SCARED) | 12893 | 0.9 | 0.66 | 1.21 | -0.11 | 0.15 | 0.48 | 0.53 |
|  | Elevated depression symptoms (SMFQ) | 12677 | 1.05 | 0.76 | 1.43 | 0.05 | 0.16 | 0.74 | 0.98 |
| Diabetes | Anxiety diagnosis | 17423 | 0.82 | 0.2 | 2.19 | -0.2 | 0.59 | 0.74 | 0.74 |
|  | MDD diagnosis | 17424 | 1.72 | 0.52 | 4.13 | 0.54 | 0.51 | 0.29 | 0.59 |
|  | Elevated anxiety symptoms (SCARED) | 13413 | 1.07 | 0.67 | 1.66 | 0.07 | 0.23 | 0.78 | 0.78 |
|  | Elevated depression symptoms (SMFQ) | 13192 | 1.04 | 0.61 | 1.68 | 0.04 | 0.26 | 0.89 | 0.98 |
| Epilepsy | Anxiety diagnosis | 17473 | 2.89 | 1.55 | 4.96 | 1.06 | 0.29 | **3.1x10^-4^** | **1.6x10^-3^** |
|  | MDD diagnosis | 17474 | 0.9 | 0.22 | 2.39 | -0.11 | 0.59 | 0.85 | 0.95 |
|  | Elevated anxiety symptoms (SCARED) | 13446 | 1.64 | 1.08 | 2.47 | 0.5 | 0.21 | **0.02** | **0.04** |
|  | Elevated depression symptoms (SMFQ) | 13225 | 1.06 | 0.63 | 1.71 | 0.06 | 0.25 | 0.81 | 0.98 |
| Migraine^ | Anxiety diagnosis | 16678 | 1.39 | 1.04 | 1.82 | 0.33 | 0.14 | **0.02** | **0.05** |
|  | MDD diagnosis | 16678 | 1.29 | 0.89 | 1.82 | 0.26 | 0.18 | 0.15 | 0.39 |
|  | Elevated anxiety symptoms (SCARED) | 12896 | 1.27 | 1.1 | 1.46 | 0.24 | 0.07 | **9.6x10^-4^** | **4.8x10^-3^** |
|  | Elevated depression symptoms (SMFQ) | 12680 | 1.36 | 1.17 | 1.58 | 0.31 | 0.08 | **6.6x10^-5^** | **3.3x10^-4^** |
| Obesity | Anxiety diagnosis | 18478 | 2.1 | 1.52 | 2.83 | 0.74 | 0.16 | **2.9x10^-6^** | **2.9x10^-5^** |
|  | MDD diagnosis | 18478 | 2.2 | 1.51 | 3.1 | 0.79 | 0.18 | **1.7x10^-5^** | **8.3x10^-5^** |
|  | Elevated anxiety symptoms (SCARED) | 18111 | 1.42 | 1.24 | 1.64 | 0.35 | 0.07 | **9.8x10^-7^** | **9.8x10^-6^** |
|  | Elevated depression symptoms (SMFQ) | 17825 | 1.72 | 1.48 | 1.99 | 0.54 | 0.07 | **4.4 x10^-13^** | **4.4x10^-12^** |
| Reduced hearing | Anxiety diagnosis | 17452 | 1.17 | 0.65 | 1.94 | 0.16 | 0.28 | 0.56 | 0.62 |
|  | MDD diagnosis | 17453 | 1.12 | 0.53 | 2.06 | 0.11 | 0.34 | 0.75 | 0.94 |
|  | Elevated anxiety symptoms (SCARED) | 13424 | 1.11 | 0.85 | 1.42 | 0.1 | 0.13 | 0.43 | 0.53 |
|  | Elevated depression symptoms (SMFQ) | 13207 | 0.94 | 0.69 | 1.25 | -0.06 | 0.15 | 0.69 | 0.98 |

**Table S5 –** Results of two-sample Mendelian Randomisation of associations between childhood LTCs and depression and anxiety outcomes in MoBa data.

| Exposure | Outcome | MR Method | N SNPs | OR | 95% CI | | P-value | P_FDR_ |
| --- | --- | --- | --- | --- | --- | --- | --- | --- |
|  |  |  |  |  | **Lower** | **Upper** |  |  |
| Juvenile arthritis | Anxiety disorder diagnosis | Inverse variance weighted | 4 | 1.01 | 0.97 | 1.06 | 0.58 | 0.76 |
|  |  | *MR Egger* | *4* | *1.06* | *0.91* | *1.23* | *0.55* |  |
|  |  | *Weighted median* | *4* | *1.02* | *0.97* | *1.08* | *0.44* |  |
|  |  | *Weighted mode* | *4* | *1.02* | *0.96* | *1.09* | *0.50* |  |
|  | MDD diagnosis | Inverse variance weighted | 4 | 0.97 | 0.90 | 1.05 | 0.47 | 0.56 |
|  |  | *MR Egger* | *4* | *0.82* | *0.69* | *0.98* | *0.16* |  |
|  |  | *Weighted median* | *4* | *0.96* | *0.90* | *1.03* | *0.29* |  |
|  |  | *Weighted mode* | *4* | *0.93* | *0.87* | *0.99* | *0.12* |  |
|  | Elevated anxiety symptoms (SCARED | Inverse variance weighted | 4 | 1.01 | 0.97 | 1.05 | 0.65 | 0.79 |
|  |  | *MR Egger* | *4* | *1.08* | *0.95* | *1.22* | *0.37* |  |
|  |  | *Weighted median* | *4* | *1.02* | *0.98* | *1.07* | *0.35* |  |
|  |  | *Weighted mode* | *4* | *1.03* | *0.98* | *1.08* | *0.38* |  |
|  | Elevated depression symptoms (SMFQ) | Inverse variance weighted | 4 | 1.00 | 0.95 | 1.06 | 0.86 | 0.86 |
|  |  | *MR Egger* | *4* | *1.00* | *0.81* | *1.23* | *0.98* |  |
|  |  | *Weighted median* | *4* | *1.00* | *0.95* | *1.05* | *0.97* |  |
|  |  | *Weighted mode* | *4* | *1.00* | *0.95* | *1.06* | *0.90* |  |
| Childhood-onset asthma | Anxiety disorder diagnosis | Inverse variance weighted | 58 | 1.11 | 1.03 | 1.21 | **0.008** | 0.05 |
|  |  | *MR Egger* | *58* | *1.10* | *0.89* | *1.36* | *0.38* |  |
|  |  | *Weighted median* | *58* | *1.07* | *0.95* | *1.21* | *0.27* |  |
|  |  | *Weighted mode* | *58* | *1.06* | *0.90* | *1.24* | *0.50* |  |
|  | MDD diagnosis | Inverse variance weighted | 58 | 1.11 | 1.02 | 1.22 | **0.02** | 0.13 |
|  |  | *MR Egger* | *58* | *1.24* | *0.98* | *1.57* | *0.08* |  |
|  |  | *Weighted median* | *58* | *1.18* | *1.03* | *1.36* | *0.02* |  |
|  |  | *Weighted mode* | *58* | *1.21* | *1.01* | *1.46* | *0.04* |  |
|  | Elevated anxiety symptoms (SCARED | Inverse variance weighted | 58 | 1.06 | 0.99 | 1.13 | 0.09 | 0.30 |
|  |  | *MR Egger* | *58* | *1.08* | *0.91* | *1.27* | *0.39* |  |
|  |  | *Weighted median* | *58* | *1.08* | *0.98* | *1.19* | *0.11* |  |
|  |  | *Weighted mode* | *58* | *1.11* | *0.97* | *1.27* | *0.15* |  |
|  | Elevated depression symptoms (SMFQ) | Inverse variance weighted | 58 | 1.01 | 0.94 | 1.09 | 0.72 | 0.84 |
|  |  | *MR Egger* | *58* | *1.02* | *0.84* | *1.23* | *0.83* |  |
|  |  | *Weighted median* | *58* | *1.04* | *0.93* | *1.16* | *0.51* |  |
|  |  | *Weighted mode* | *58* | *1.01* | *0.88* | *1.16* | *0.86* |  |
| Type 1 diabetes | Anxiety disorder diagnosis | Inverse variance weighted | 25 | 1.03 | 0.99 | 1.08 | 0.10 | 0.36 |
|  |  | *MR Egger* | *25* | *1.00* | *0.95* | *1.06* | *0.93* |  |
|  |  | *Weighted median* | *25* | *1.02* | *0.97* | *1.07* | *0.45* |  |
|  |  | *Weighted mode* | *25* | *1.01* | *0.97* | *1.06* | *0.56* |  |
|  | MDD diagnosis | Inverse variance weighted | 25 | 1.03 | 0.97 | 1.09 | 0.29 | 0.56 |
|  |  | *MR Egger* | *25* | *1.04* | *0.96* | *1.13* | *0.35* |  |
|  |  | *Weighted median* | *25* | *1.04* | *0.99* | *1.10* | *0.14* |  |
|  |  | *Weighted mode* | *25* | *1.04* | *0.99* | *1.10* | *0.17* |  |
|  | Elevated anxiety symptoms (SCARED | Inverse variance weighted | 25 | 1.00 | 0.97 | 1.04 | 0.79 | 0.79 |
|  |  | *MR Egger* | *25* | *1.03* | *0.98* | *1.08* | *0.19* |  |
|  |  | *Weighted median* | *25* | *1.03* | *0.99* | *1.07* | *0.13* |  |
|  |  | *Weighted mode* | *25* | *1.03* | *0.99* | *1.08* | *0.16* |  |
|  | Elevated depression symptoms (SMFQ) | Inverse variance weighted | 25 | 0.99 | 0.95 | 1.03 | 0.51 | 0.72 |
|  |  | *MR Egger* | *25* | *1.01* | *0.95* | *1.06* | *0.82* |  |
|  |  | *Weighted median* | *25* | *1.01* | *0.96* | *1.05* | *0.78* |  |
|  |  | *Weighted mode* | *25* | *1.00* | *0.96* | *1.04* | *0.96* |  |
| Eczema | Anxiety disorder diagnosis | Inverse variance weighted | 9 | 1.04 | 0.88 | 1.23 | 0.66 | 0.77 |
|  |  | *MR Egger* | *9* | *0.85* | *0.44* | *1.66* | *0.66* |  |
|  |  | *Weighted median* | *9* | *1.05* | *0.85* | *1.31* | *0.62* |  |
|  |  | *Weighted mode* | *9* | *1.09* | *0.8* | *1.49* | *0.61* |  |
|  | MDD diagnosis | Inverse variance weighted | 9 | 0.94 | 0.77 | 1.15 | 0.57 | 0.57 |
|  |  | *MR Egger* | *9* | *0.80* | *0.36* | *1.77* | *0.61* |  |
|  |  | *Weighted median* | *9* | *0.90* | *0.70* | *1.16* | *0.43* |  |
|  |  | *Weighted mode* | *9* | *0.86* | *0.61* | *1.23* | *0.44* |  |
|  | Elevated anxiety symptoms (SCARED | Inverse variance weighted | 9 | 0.95 | 0.77 | 1.17 | 0.62 | 0.79 |
|  |  | *MR Egger* | *9* | *1.00* | *0.42* | *2.41* | *1.00* |  |
|  |  | *Weighted median* | *9* | *1.01* | *0.83* | *1.23* | *0.90* |  |
|  |  | *Weighted mode* | *9* | *0.98* | *0.77* | *1.24* | *0.88* |  |
|  | Elevated depression symptoms (SMFQ) | Inverse variance weighted | 9 | 1.10 | 0.90 | 1.34 | 0.34 | 0.63 |
|  |  | *MR Egger* | *9* | *0.99* | *0.43* | *2.30* | *0.98* |  |
|  |  | *Weighted median* | *9* | *1.05* | *0.85* | *1.31* | *0.64* |  |
|  |  | *Weighted mode* | *9* | *1.09* | *0.85* | *1.40* | *0.52* |  |
| Epilepsy | Anxiety disorder diagnosis | Inverse variance weighted | 16 | 0.91 | 0.70 | 1.17 | 0.45 | 0.77 |
|  |  | *MR Egger* | *16* | *1.06* | *0.28* | *3.94* | *0.93* |  |
|  |  | *Weighted median* | *16* | *0.92* | *0.65* | *1.29* | *0.63* |  |
|  |  | *Weighted mode* | *16* | *0.98* | *0.54* | *1.79* | *0.95* |  |
|  | MDD diagnosis | Inverse variance weighted | 16 | 0.84 | 0.61 | 1.17 | 0.31 | 0.56 |
|  |  | *MR Egger* | *16* | *0.66* | *0.11* | *3.93* | *0.66* |  |
|  |  | *Weighted median* | *16* | *0.83* | *0.55* | *1.25* | *0.37* |  |
|  |  | *Weighted mode* | *16* | *0.77* | *0.37* | *1.58* | *0.49* |  |
|  | Elevated anxiety symptoms (SCARED | Inverse variance weighted | 16 | 1.03 | 0.83 | 1.28 | 0.77 | 0.79 |
|  |  | *MR Egger* | *16* | *1.01* | *0.34* | *2.99* | *0.99* |  |
|  |  | *Weighted median* | *16* | *1.00* | *0.75* | *1.34* | *0.99* |  |
|  |  | *Weighted mode* | *16* | *0.96* | *0.60* | *1.55* | *0.87* |  |
|  | Elevated depression symptoms (SMFQ) | Inverse variance weighted | 16 | 0.89 | 0.70 | 1.12 | 0.31 | 0.63 |
|  |  | *MR Egger* | *16* | *1.73* | *0.53* | *5.62* | *0.38* |  |
|  |  | *Weighted median* | *16* | *0.84* | *0.62* | *1.15* | *0.28* |  |
|  |  | *Weighted mode* | *16* | *0.87* | *0.51* | *1.47* | *0.60* |  |
| Migraine | Anxiety disorder diagnosis | Inverse variance weighted | 81 | 1.07 | 0.91 | 1.26 | 0.43 | 0.77 |
|  |  | *MR Egger* | *81* | *0.93* | *0.62* | *1.41* | *0.73* |  |
|  |  | *Weighted median* | *81* | *1.00* | *0.77* | *1.31* | *0.99* |  |
|  |  | *Weighted mode* | *81* | *1.04* | *0.69* | *1.58* | *0.84* |  |
|  | MDD diagnosis | Inverse variance weighted | 81 | 1.09 | 0.90 | 1.33 | 0.39 | 0.56 |
|  |  | *MR Egger* | *81* | *1.39* | *0.85* | *2.26* | *0.19* |  |
|  |  | *Weighted median* | *81* | *1.24* | *0.93* | *1.66* | *0.14* |  |
|  |  | *Weighted mode* | *81* | *1.28* | *0.86* | *1.90* | *0.23* |  |
|  | Elevated anxiety symptoms (SCARED | Inverse variance weighted | 81 | 1.20 | 1.04 | 1.38 | **0.01** | 0.08 |
|  |  | *MR Egger* | *81* | *1.15* | *0.81* | *1.62* | *0.44* |  |
|  |  | *Weighted median* | *81* | *1.16* | *0.94* | *1.43* | *0.16* |  |
|  |  | *Weighted mode* | *81* | *1.21* | *0.88* | *1.68* | *0.25* |  |
|  | Elevated depression symptoms (SMFQ) | Inverse variance weighted | 81 | 1.38 | 1.19 | 1.60 | **2.6x10^-5^** | **1.8x10^-4^** |
|  |  | *MR Egger* | *81* | *1.54* | *1.06* | *2.22* | ***0.02*** |  |
|  |  | *Weighted median* | *81* | *1.48* | *1.19* | *1.84* | ***2.6x10^-5^*** |  |
|  |  | *Weighted mode* | *81* | *1.53* | *1.16* | *2.00* | ***3.0x10^-3^*** |  |
| Childhood obesity | Anxiety disorder diagnosis | Inverse variance weighted | 7 | 1.00 | 0.81 | 1.22 | 0.96 | 0.96 |
|  |  | *MR Egger* | *7* | *0.91* | *0.27* | *3.08* | *0.89* |  |
|  |  | *Weighted median* | *7* | *0.93* | *0.77* | *1.13* | *0.49* |  |
|  |  | *Weighted mode* | *7* | *0.99* | *0.74* | *1.32* | *0.94* |  |
|  | MDD diagnosis | Inverse variance weighted | 7 | 0.92 | 0.73 | 1.16 | 0.48 | 0.56 |
|  |  | *MR Egger* | *7* | *2.6* | *0.87* | *7.81* | *0.15* |  |
|  |  | *Weighted median* | *7* | *0.93* | *0.73* | *1.19* | *0.59* |  |
|  |  | *Weighted mode* | *7* | *1.01* | *0.71* | *1.43* | *0.97* |  |
|  | Elevated anxiety symptoms (SCARED | Inverse variance weighted | 7 | 0.91 | 0.76 | 1.09 | 0.30 | 0.70 |
|  |  | *MR Egger* | *7* | *1.57* | *0.60* | *4.11* | *0.40* |  |
|  |  | *Weighted median* | *7* | *1.00* | *0.84* | *1.19* | *0.99* |  |
|  |  | *Weighted mode* | *7* | *1.04* | *0.87* | *1.24* | *0.67* |  |
|  | Elevated depression symptoms (SMFQ) | Inverse variance weighted | 7 | 1.07 | 0.93 | 1.23 | 0.36 | 0.63 |
|  |  | *MR Egger* | *7* | *0.91* | *0.39* | *2.14* | *0.83* |  |
|  |  | *Weighted median* | *7* | *1.06* | *0.88* | *1.28* | *0.54* |  |
|  |  | *Weighted mode* | *7* | *1.07* | *0.84* | *1.37* | *0.61* |  |

**Table S6 -** Results from MR Egger testing for horizontal pleiotropy in the MR analysis between childhood LTCs and anxiety and depression outcomes using the MoBa data.

| Exposure | Outcome | Egger intercept | SE | P-value |
| --- | --- | --- | --- | --- |
| Childhood obesity | Anxiety diagnosis | 0.01 | 0.11 | 0.89 |
|  | MDD diagnosis | -0.18 | 0.10 | 0.12 |
|  | Elevated anxiety symptoms (SCARED) | -0.10 | 0.08 | 0.31 |
|  | Elevated depression symptoms (SMFQ) | 0.03 | 0.07 | 0.72 |
| Childhood-onset asthma | Anxiety diagnosis | 0.002 | 0.01 | 0.89 |
|  | MDD diagnosis | -0.01 | 0.01 | 0.35 |
|  | Elevated anxiety symptoms (SCARED) | -0.002 | 0.01 | 0.82 |
|  | Elevated depression symptoms (SMFQ) | -0.0008 | 0.01 | 0.94 |
| Eczema | Anxiety diagnosis | 0.03 | 0.04 | 0.57 |
|  | MDD diagnosis | 0.02 | 0.05 | 0.69 |
|  | Elevated anxiety symptoms (SCARED) | -0.01 | 0.06 | 0.91 |
|  | Elevated depression symptoms (SMFQ) | 0.01 | 0.06 | 0.80 |
| Genetic generalised epilepsy | Anxiety diagnosis | -0.01 | 0.04 | 0.82 |
|  | MDD diagnosis | 0.02 | 0.06 | 0.79 |
|  | Elevated anxiety symptoms (SCARED) | 0.00 | 0.04 | 0.96 |
|  | Elevated depression symptoms (SMFQ) | -0.05 | 0.04 | 0.28 |
| Juvenile idiopathic arthritis | Anxiety diagnosis | -0.03 | 0.06 | 0.63 |
|  | MDD diagnosis | 0.13 | 0.07 | 0.19 |
|  | Elevated anxiety symptoms (SCARED) | -0.05 | 0.05 | 0.41 |
|  | Elevated depression symptoms (SMFQ) | 0.01 | 0.08 | 0.95 |
| Migraine | Anxiety diagnosis | 0.01 | 0.01 | 0.47 |
|  | MDD diagnosis | -0.01 | 0.01 | 0.29 |
|  | Elevated anxiety symptoms (SCARED) | 0.00 | 0.01 | 0.78 |
|  | Elevated depression symptoms (SMFQ) | -0.01 | 0.01 | 0.52 |
| Type 1 diabetes | Anxiety diagnosis | 0.02 | 0.01 | 0.15 |
|  | MDD diagnosis | -0.01 | 0.02 | 0.74 |
|  | Elevated anxiety symptoms (SCARED) | -0.02 | 0.01 | 0.12 |
|  | Elevated depression symptoms (SMFQ) | -0.01 | 0.01 | 0.35 |

**Table S7 –** Results from MR-PRESSO tests for nominally significant causal relationships using MoBa mental health outcomes. The first step of MR-PRESSO (global test) indicated no significant effect of horizontal pleiotropy, therefore no further steps (outlier corrected causal estimate and distortion test) were not carried out.

| Exposure | Outcome | Global test | |
| --- | --- | --- | --- |
|  |  | **RSSobs** | **P-value** |
| Migraine | Elevated anxiety symptoms (SCARED) | 92.33 | 0.21 |
|  | Elevated depression symptoms (SMFQ) | 86.84 | 0.34 |
| Childhood-onset asthma | Anxiety diagnosis | 64.86 | 0.29 |
|  | MDD diagnosis | 59.31 | 0.46 |

**Table S8** – Heterogeneity statistics for MR using MoBa outcome data.

| Exposure | Outcome | Method | Q | DF | P-value |
| --- | --- | --- | --- | --- | --- |
| Childhood obesity | Anxiety diagnosis | MR Egger | 11.2 | 5 | 0.05 |
|  |  | Inverse variance weighted | 11.2 | 6 | 0.08 |
|  | MDD diagnosis | MR Egger | 6.4 | 5 | 0.27 |
|  |  | Inverse variance weighted | 11.0 | 6 | 0.09 |
|  | Elevated anxiety symptoms (SCARED) | MR Egger | 9.8 | 5 | 0.08 |
|  |  | Inverse variance weighted | 12.3 | 6 | 0.06 |
|  | Elevated depression symptoms (SMFQ) | MR Egger | 6.6 | 5 | 0.25 |
|  |  | Inverse variance weighted | 6.8 | 6 | 0.34 |
| Childhood-onset asthma | Anxiety diagnosis | MR Egger | 62.8 | 56 | 0.25 |
|  |  | Inverse variance weighted | 62.8 | 57 | 0.28 |
|  | MDD diagnosis | MR Egger | 56.6 | 56 | 0.45 |
|  |  | Inverse variance weighted | 57.5 | 57 | 0.46 |
|  | Elevated anxiety symptoms (SCARED) | MR Egger | 52.0 | 56 | 0.63 |
|  |  | Inverse variance weighted | 52.0 | 57 | 0.66 |
|  | Elevated depression symptoms (SMFQ) | MR Egger | 60.3 | 56 | 0.32 |
|  |  | Inverse variance weighted | 60.3 | 57 | 0.36 |
| Eczema | Anxiety diagnosis | MR Egger | 5.3 | 7 | 0.63 |
|  |  | Inverse variance weighted | 5.6 | 8 | 0.69 |
|  | MDD diagnosis | MR Egger | 4.7 | 7 | 0.70 |
|  |  | Inverse variance weighted | 4.8 | 8 | 0.78 |
|  | Elevated anxiety symptoms (SCARED) | MR Egger | 16.7 | 7 | **0.02** |
|  |  | Inverse variance weighted | 16.7 | 8 | **0.03** |
|  | Elevated depression symptoms (SMFQ) | MR Egger | 13.1 | 7 | 0.07 |
|  |  | Inverse variance weighted | 13.3 | 8 | 0.10 |
| Epilepsy | Anxiety diagnosis | MR Egger | 14.4 | 14 | 0.42 |
|  |  | Inverse variance weighted | 14.4 | 15 | 0.49 |
|  | MDD diagnosis | MR Egger | 17.6 | 14 | 0.23 |
|  |  | Inverse variance weighted | 17.7 | 15 | 0.28 |
|  | Elevated anxiety symptoms (SCARED) | MR Egger | 9.6 | 14 | 0.79 |
|  |  | Inverse variance weighted | 9.6 | 15 | 0.84 |
|  | Elevated depression symptoms (SMFQ) | MR Egger | 13.0 | 14 | 0.53 |
|  |  | Inverse variance weighted | 14.2 | 15 | 0.51 |
| Juvenile idiopathic arthritis | Anxiety diagnosis | MR Egger | 1.2 | 2 | 0.56 |
|  |  | Inverse variance weighted | 1.5 | 3 | 0.69 |
|  | MDD diagnosis | MR Egger | 1.4 | 2 | 0.51 |
|  |  | Inverse variance weighted | 5.3 | 3 | 0.15 |
|  | Elevated anxiety symptoms (SCARED) | MR Egger | 1.5 | 2 | 0.47 |
|  |  | Inverse variance weighted | 2.6 | 3 | 0.46 |
|  | Elevated depression symptoms (SMFQ) | MR Egger | 4.7 | 2 | 0.10 |
|  |  | Inverse variance weighted | 4.7 | 3 | 0.20 |
| Migraine | Anxiety diagnosis | MR Egger | 90.1 | 79 | 0.18 |
|  |  | Inverse variance weighted | 90.7 | 80 | 0.19 |
|  | MDD diagnosis | MR Egger | 89.6 | 79 | 0.19 |
|  |  | Inverse variance weighted | 90.9 | 80 | 0.19 |
|  | Elevated anxiety symptoms (SCARED) | MR Egger | 89.9 | 79 | 0.19 |
|  |  | Inverse variance weighted | 90.0 | 80 | 0.21 |
|  | Elevated depression symptoms (SMFQ) | MR Egger | 84.7 | 79 | 0.31 |
|  |  | Inverse variance weighted | 85.2 | 80 | 0.33 |
| Type 1 diabetes | Anxiety diagnosis | MR Egger | 23.5 | 23 | 0.43 |
|  |  | Inverse variance weighted | 25.7 | 24 | 0.37 |
|  | MDD diagnosis | MR Egger | 36.2 | 23 | **0.04** |
|  |  | Inverse variance weighted | 36.3 | 24 | 0.05 |
|  | Elevated anxiety symptoms (SCARED) | MR Egger | 23.0 | 23 | 0.46 |
|  |  | Inverse variance weighted | 25.5 | 24 | 0.38 |
|  | Elevated depression symptoms (SMFQ) | MR Egger | 24.3 | 23 | 0.39 |
|  |  | Inverse variance weighted | 25.3 | 24 | 0.39 |

**Table S9** – Details instrument F statistics across all exposures

| Exposure | F statistic  (mean across all instruments) | Minimum F | N instruments |
| --- | --- | --- | --- |
| Direction LTC -> dep/anx | | | |
| Childhood obesity | 59.5 | 30.5 | 8 |
| Childhood-onset asthma | 83.9 | 23.6 | 86 |
| Eczema | 47.8 | 29.9 | 10 |
| Epilepsy | 41.7 | 29.7 | 19 |
| Juvenile arthritis | 118.1 | 54.5 | 4 |
| Migraine | 59.8 | 29.8 | 96 |
| Type 1 diabetes | 115.7 | 30.5 | 40 |
| Direction Dep -> LTC | | | |
| iPSYCH youth-onset depression | 23.5 | 21.0 | 15 |

**Table S10** – Steiger directionality tests for potentially causal links between LTCs and anxiety/depression outcomes in MoBa MR analyses.

| Exposure | Outcome | R2 exposure | R2 outcome | Steiger P-value | Correct causal direction? |
| --- | --- | --- | --- | --- | --- |
| Migraine | Elevated anxiety symptoms (SCARED) | 0.022 | 0.00092 | 1.2x10^-30^ | TRUE |
|  | Elevated depression symptoms (SMFQ) | 0.022 | 0.00066 | 8.8x10^-33^ | TRUE |
| Childhood-onset asthma | Anxiety diagnosis | 0.017 | 0.0011 | 1.2x10^-126^ | TRUE |
|  | MDD diagnosis | 0.017 | 0.00086 | 2.8x10^-135^ | TRUE |

**Table S11 –** Two-sample Mendelian Randomisation analyses of associations between youth-onset depression and childhood LTCs (alternate direction to Table 2 in main text). Note that MR associations could not be estimated for genetic generalised epilepsy in this direction due to genetic instruments absent in available data and no appropriate proxies identified.

| Exposure | Outcome | MR Method | N SNPs | OR | 95% CI | | P-value |
| --- | --- | --- | --- | --- | --- | --- | --- |
|  |  |  |  |  | **Lower** | **Upper** |  |
| Youth-onset depression | Juvenile idiopathic arthritis | Wald ratio | 1 | 0.89 | 0.39 | 2.02 | 0.78 |
| Youth-onset depression | Childhood-onset asthma | Inverse variance weighted | 15 | 1.00 | 0.94 | 1.06 | 0.97 |
|  |  | *MR Egger* | *15* | *1.02* | *0.91* | *1.15* | *0.76* |
|  |  | *Weighted median* | *15* | *1.00* | *0.93* | *1.08* | *0.95* |
|  |  | *Weighted mode* | *15* | *1.00* | *0.87* | *1.14* | *0.98* |
| Youth-onset depression | Type 1 diabetes | Inverse variance weighted | 15 | 1.01 | 0.88 | 1.15 | 0.91 |
|  |  | *MR Egger* | *15* | *1.26* | *0.98* | *1.61* | *0.10* |
|  |  | *Weighted median* | *15* | *0.96* | *0.82* | *1.14* | *0.67* |
|  |  | *Weighted mode* | *15* | *0.95* | *0.76* | *1.18* | *0.66* |
| Youth-onset depression | Eczema | Inverse variance weighted | 15 | 1.01 | 0.90 | 1.15 | 0.82 |
|  |  | *MR Egger* | *15* | *1.11* | *0.83* | *1.49* | *0.48* |
|  |  | *Weighted median* | *15* | *0.90* | *0.78* | *1.04* | *0.16* |
|  |  | *Weighted mode* | *15* | *0.89* | *0.75* | *1.07* | *0.24* |
| Youth-onset depression | Migraine | Inverse variance weighted | 15 | 1.00 | 0.96 | 1.04 | 0.92 |
|  |  | *MR Egger* | *15* | *0.98* | *0.89* | *1.09* | *0.76* |
|  |  | *Weighted median* | *15* | *0.99* | *0.93* | *1.04* | *0.63* |
|  |  | *Weighted mode* | *15* | *0.99* | *0.91* | *1.09* | *0.91* |
| Youth-onset depression | Childhood obesity | Inverse variance weighted | 13 | 1.00 | 0.89 | 1.12 | 0.97 |
|  |  | *MR Egger* | *13* | *0.73* | *0.56* | *0.96* | *0.05* |
|  |  | *Weighted median* | *13* | *1.00* | *0.85* | *1.17* | *0.95* |
|  |  | *Weighted mode* | *13* | *1.03* | *0.82* | *1.29* | *0.79* |

**Table S12 –** Results from MR Egger testing for horizontal pleiotropy in the two-sample bidirectional MR of LTCs on youth-onset depression using GWAS summary statistics.

| Exposure | Outcome | Egger intercept | SE | P-value |
| --- | --- | --- | --- | --- |
| Childhood obesity | Youth-onset depression | 0.019 | 0.041 | 0.66 |
| Childhood-onset asthma | Youth-onset depression | 0.004 | 0.006 | 0.49 |
| Eczema | Youth-onset depression | -0.027 | 0.034 | 0.45 |
| Genetic generalised epilepsy | Youth-onset depression | 0.023 | 0.039 | 0.56 |
| Juvenile idiopathic arthritis | Youth-onset depression | -0.057 | 0.036 | 0.26 |
| Migraine | Youth-onset depression | 0.012 | 0.006 | 0.05 |
| Type 1 diabetes | Youth-onset depression | 0.010 | 0.007 | 0.13 |
| Youth-onset depression | Childhood obesity | 0.057 | 0.023 | 0.03 |
| Youth-onset depression | Childhood-onset asthma | -0.004 | 0.012 | 0.71 |
| Youth-onset depression | Eczema | -0.017 | 0.025 | 0.50 |
| Youth-onset depression | Juvenile idiopathic arthritis | - | - | - |
| Youth-onset depression | Migraine | 0.003 | 0.009 | 0.77 |
| Youth-onset depression | Type 1 diabetes | -0.049 | 0.025 | 0.07 |

**Table S13** – Heterogeneity statistics for bidirectional MR using iPSYCH early-onset depression GWAS summary data.

| Exposure | Outcome | MR Method | Q | DF | P-value |
| --- | --- | --- | --- | --- | --- |
| Childhood obesity | Youth-onset depression | MR Egger | 7.87 | 6 | 0.25 |
|  |  | Inverse variance weighted | 8.16 | 7 | 0.32 |
| Childhood-onset asthma |  | MR Egger | 63.80 | 84 | 0.95 |
|  |  | Inverse variance weighted | 64.28 | 85 | 0.95 |
| Eczema |  | MR Egger | 10.89 | 8 | 0.21 |
|  |  | Inverse variance weighted | 11.75 | 9 | 0.23 |
| Epilepsy |  | MR Egger | 30.52 | 17 | **0.02** |
|  |  | Inverse variance weighted | 31.15 | 18 | **0.03** |
| Migraine |  | MR Egger | 93.50 | 94 | 0.50 |
|  |  | Inverse variance weighted | 97.47 | 95 | 0.41 |
| Type 1 diabetes |  | MR Egger | 34.66 | 38 | 0.62 |
|  |  | Inverse variance weighted | 37.11 | 39 | 0.56 |
| Juvenile idiopathic arthritis |  | MR Egger | 1.00 | 2 | 0.61 |
|  |  | Inverse variance weighted | 3.48 | 3 | 0.32 |
| Youth-onset depression | Childhood obesity | MR Egger | 6.60 | 11 | 0.83 |
|  |  | Inverse variance weighted | 12.58 | 12 | 0.40 |
|  | Childhood-onset asthma | MR Egger | 8.12 | 13 | 0.84 |
|  |  | Inverse variance weighted | 8.26 | 14 | 0.88 |
|  | Eczema | MR Egger | 17.40 | 13 | 0.18 |
|  |  | Inverse variance weighted | 18.03 | 14 | 0.21 |
|  | Migraine | MR Egger | 13.80 | 13 | 0.39 |
|  |  | Inverse variance weighted | 13.89 | 14 | 0.46 |
|  | Type 1 diabetes | MR Egger | 14.30 | 13 | 0.35 |
|  |  | Inverse variance weighted | 18.53 | 14 | 0.18 |

**Table S14** – Phenotypic associations between childhood long-term physical health conditions (LTCs) and subsequent anxiety/depression diagnosis (aged 14-18 years).

| LTC | N | OR | 95%CI | | Beta | SE | P-value | P_FDR_ |
| --- | --- | --- | --- | --- | --- | --- | --- | --- |
|  |  |  | **Lower** | **Upper** |  |  |  |  |
| Anxiety diagnosis | | | | | | | |  |
| Any LTC | 17381 | 1.37 | 1.09 | 1.71 | 0.31 | 0.12 | **0.006** | **-** |
| Arthritis | 17269 | 3.79 | 1.13 | 9.49 | 1.33 | 0.53 | **0.012** | 0.06 |
| Asthma^ | 16466 | 1.07 | 0.75 | 1.49 | 0.07 | 0.18 | 0.70 | 0.94 |
| Cerebral palsy | 40721 | 3.63 | 0.92 | 10.63 | 1.34 | 0.6 | **0.026** | 0.09 |
| Chronic fatigue^ | 16509 | 2.36 | 0.57 | 6.59 | 0.86 | 0.6 | 0.16 | 0.26 |
| Coeliac^ | 16494 | 0.98 | 0.38 | 2.04 | -0.00 | 0.42 | 0.96 | 0.96 |
| Diabetes | 17239 | 0.87 | 0.14 | 2.76 | -0.10 | 0.72 | 0.84 | 0.94 |
| Epilepsy | 17268 | 1.99 | 0.77 | 4.19 | 0.69 | 0.42 | 0.11 | 0.21 |
| Migraine^ | 16507 | 1.4 | 0.97 | 1.95 | 0.34 | 0.18 | 0.057 | 0.14 |
| Obesity | 18301 | 2.6 | 1.79 | 3.66 | 0.95 | 0.18 | **1.7x10^-7^** | **1.7x10^-6^** |
| Reduced hearing | 17269 | 0.9 | 0.38 | 1.78 | -0.10 | 0.39 | 0.79 | 0.94 |
| Depression diagnosis | | | | | | | |  |
| Any LTC | 17499 | 1.55 | 1.21 | 1.97 | 0.44 | 0.12 | **4.7x10^-4^** | **-** |
| Arthritis | 17411 | 0.00 | 0.00 | 0.74 | -12.0 | 308.6 | 0.97 | 0.97 |
| Asthma^ | 16595 | 1.38 | 0.96 | 1.94 | 0.33 | 0.18 | 0.067 | 0.22 |
| Cerebral palsy | 41044 | 1.34 | 0.08 | 6.21 | 0.29 | 1.02 | 0.77 | 0.97 |
| Chronic fatigue^ | 16620 | 5.27 | 1.79 | 12.43 | 1.66 | 0.48 | **0.001** | **0.006** |
| Coeliac^ | 16605 | 0.8 | 0.24 | 1.9 | -0.2 | 0.51 | 0.66 | 0.94 |
| Diabetes | 17360 | 1.61 | 0.39 | 4.33 | 0.48 | 0.59 | 0.42 | 0.70 |
| Epilepsy | 17410 | 0.35 | 0.02 | 1.58 | -1.1 | 1.01 | 0.30 | 0.59 |
| Migraine^ | 16618 | 1.33 | 0.88 | 1.93 | 0.28 | 0.2 | 0.15 | 0.38 |
| Obesity | 18417 | 1.96 | 1.27 | 2.9 | 0.67 | 0.21 | **0.001** | **0.007** |
| Reduced hearing | 17369 | 1.06 | 0.45 | 2.1 | 0.06 | 0.39 | 0.88 | 0.97 |

*^Indicates models where sex was included as a covariate.*

**Table S15 –** Phenotypic associations between childhood LTC and total depression (SMFQ) and anxiety (SCARED) symptom scores**.**

| LTC | N | Beta | SE | P-value | P_FDR_ |  |
| --- | --- | --- | --- | --- | --- | --- |
| Anxiety symptoms | | | | | | |
| Any LTC | 13539 | 0.23 | 0.04 | **3.8x10^-10^** | - |  |
| Arthritis | 13444 | 0.517 | 0.302 | 0.087 | 0.14 |  |
| Asthma^ | 12880 | 0.134 | 0.048 | **0.0054** | **0.014** |  |
| Cerebral palsy | 13485 | 1.365 | 0.428 | **0.0015** | **0.005** |  |
| Chronic fatigue^ | 12899 | 0.466 | 0.305 | 0.13 | 0.18 |  |
| Coeliac^ | 12893 | -0.06 | 0.116 | 0.60 | 0.61 |  |
| Diabetes | 13413 | -0.121 | 0.195 | 0.54 | 0.61 |  |
| Epilepsy | 13446 | 0.337 | 0.192 | 0.080 | 0.14 |  |
| Migraine^ | 12896 | 0.29 | 0.056 | **2.1x10^-7^** | **1.1x10^-6^** |  |
| Obesity | 18111 | 0.432 | 0.065 | **3.3x10^-11^** | **3.3x10^-10^** |  |
| Reduced hearing | 13424 | 0.057 | 0.111 | 0.61 | 0.61 |  |
| Depression symptoms | | | | | | |
| Any LTC | 13314 | 0.79 | 0.12 | **2.5x10^-11^** | **-** |  |
| Arthritis | 13222 | 2.151 | 0.946 | **0.023** | 0.06 |  |
| Asthma^ | 12665 | 0.498 | 0.155 | **0.0013** | **0.004** |  |
| Cerebral palsy | 13343 | 1.75 | 1.487 | 0.24 | 0.37 |  |
| Chronic fatigue^ | 12682 | 1.305 | 0.932 | 0.16 | 0.32 |  |
| Coeliac^ | 12677 | -0.414 | 0.375 | 0.27 | 0.37 |  |
| Diabetes | 13192 | -0.155 | 0.636 | 0.81 | 0.81 |  |
| Epilepsy | 13225 | 0.648 | 0.623 | 0.30 | 0.37 |  |
| Obesity | 17825 | 1.712 | 0.210 | **4.4x10^-16^** | **4.4x10^-15^** |  |
| Migraine^ | 12680 | 0.693 | 0.18 | **0.00012** | **0.0004** |  |
| Reduced hearing | 13207 | -0.086 | 0.359 | 0.81 | 0.81 |  |

*^Indicates models where sex was included as a covariate.*

**Table S16** - Phenotypic associations between childhood LTCs and parent-reported depressive symptom scores at age 14.

| LTC | N | Beta | SE | P | P_FDR_ |
| --- | --- | --- | --- | --- | --- |
| Any LTC | 16790 | 0.34 | 0.032 | **1.4x10^-25^** | **-** |
| Arthritis | 17041 | 0.152 | 0.246 | 0.54 | 0.54 |
| Asthma^ | 16244 | 0.227 | 0.044 | **2.1x10^-7^** | **4.1x10^-7^** |
| Cerebral palsy | 10145 | 0.733 | 0.421 | 0.082 | 0.10 |
| Chronic fatigue syndrome^ | 16266 | 2.064 | 0.239 | **7.1x10^-18^** | **4.9x10^-17^** |
| Coeliac^ | 16252 | 0.084 | 0.105 | 0.42 | 0.47 |
| Diabetes | 16993 | 0.418 | 0.168 | **0.013** | **0.02** |
| Epilepsy | 17042 | 1 | 0.154 | **8.6x10^-11^** | **2.2x10^-10^** |
| Migraine^ | 16264 | 0.35 | 0.05 | **3.4x10^-12^** | **1.1x10^-11^** |
| Obesity | 11026 | 0.68 | 0.079 | **9.7x10^-18^** | **4.9x10^-17^** |
| Reduced hearing | 17019 | 0.311 | 0.093 | **0.00085** | **0.001** |

*^Indicates models where sex was included as a covariate.*

**Table S17** –Two-sample MR results between childhood LTCs (GWASs used outlined in main text Table 1) and depression/anxiety (Als *et al.* (2023)) and Purves *et al.* (2020)).

| Exposure | Outcome | MR Method | N SNPs | OR | 95% CI | | P-value | P_FDR_ |
| --- | --- | --- | --- | --- | --- | --- | --- | --- |
|  |  |  |  |  | **Lower** | **Upper** |  |  |
| Juvenile idiopathic arthritis | Depression  (Als et al. 2023) | Inverse variance weighted | 4 | 1.00 | 0.99 | 1.01 | 0.72 | 0.94 |
|  |  | *MR Egger* | 4 | 0.98 | 0.97 | 1.00 | 0.17 |  |
|  |  | *Weighted median* | 4 | 1.00 | 0.99 | 1.00 | 0.66 |  |
|  |  | *Weighted mode* | 4 | 1.00 | 0.99 | 1.00 | 0.22 |  |
|  | Anxiety  (Purves et al. 2022) | Wald ratio | 1 | 0.95 | 0.89 | 1.02 | 0.19 | 0.67 |
| Childhood-onset asthma | Depression (Als et al. 2023) | Inverse variance weighted | 85 | 1.00 | 0.99 | 1.01 | 0.58 | 0.92 |
|  |  | *MR Egger* | 85 | 1.00 | 0.98 | 1.03 | 0.86 |  |
|  |  | *Weighted median* | 85 | 1.01 | 0.99 | 1.02 | 0.49 |  |
|  |  | *Weighted mode* | 85 | 1.01 | 0.99 | 1.03 | 0.39 |  |
|  | Anxiety (Purves et al. 2022) | Inverse variance weighted | 71 | 1.00 | 0.96 | 1.03 | 0.80 | 1.00 |
|  |  | *MR Egger* | 71 | 1.00 | 0.92 | 1.08 | 0.99 |  |
|  |  | *Weighted median* | 71 | 0.99 | 0.94 | 1.04 | 0.77 |  |
|  |  | *Weighted mode* | 71 | 1.00 | 0.94 | 1.07 | 0.91 |  |
| Type 1 diabetes | Depression (Als et al. 2023) | Inverse variance weighted | 37 | 1.00 | 0.99 | 1.00 | 0.38 | 0.76 |
|  |  | *MR Egger* | 37 | 1.00 | 0.99 | 1.01 | 0.83 |  |
|  |  | *Weighted median* | 37 | 1.00 | 0.99 | 1.00 | 0.73 |  |
|  |  | *Weighted mode* | 37 | 1.00 | 0.99 | 1.00 | 0.48 |  |
|  | Anxiety (Purves et al. 2022) | Inverse variance weighted | 20 | 0.97 | 0.93 | 1.00 | **0.03** | 0.25 |
|  |  | *MR Egger* | 20 | 0.96 | 0.89 | 1.03 | 0.26 |  |
|  |  | *Weighted median* | 20 | 0.96 | 0.92 | 1.01 | 0.10 |  |
|  |  | *Weighted mode* | 20 | 0.96 | 0.91 | 1.01 | 0.16 |  |
| Eczema | Depression (Als et al. 2023) | Inverse variance weighted | 10 | 1.02 | 0.99 | 1.04 | 0.15 | 0.39 |
|  |  | *MR Egger* | 10 | 0.98 | 0.90 | 1.07 | 0.74 |  |
|  |  | *Weighted median* | 10 | 1.01 | 0.99 | 1.04 | 0.22 |  |
|  |  | *Weighted mode* | 10 | 1.02 | 0.99 | 1.04 | 0.30 |  |
|  | Anxiety (Purves et al. 2022) | Inverse variance weighted | 9 | 1.01 | 0.93 | 1.10 | 0.79 | 1.00 |
|  |  | *MR Egger* | 9 | 0.99 | 0.68 | 1.42 | 0.94 |  |
|  |  | *Weighted median* | 9 | 1.00 | 0.90 | 1.11 | 1.00 |  |
|  |  | *Weighted mode* | 9 | 1.01 | 0.88 | 1.16 | 0.86 |  |
| Migraine | Depression (Als et al. 2023) | Inverse variance weighted | 96 | 1.04 | 1.00 | 1.07 | **0.03** | 0.21 |
|  |  | *MR Egger* | 96 | 0.95 | 0.88 | 1.04 | 0.26 |  |
|  |  | *Weighted median* | 96 | 1.02 | 0.99 | 1.04 | 0.25 |  |
|  |  | *Weighted mode* | 96 | 1.00 | 0.97 | 1.04 | 0.96 |  |
|  | Anxiety (Purves et al. 2022) | Inverse variance weighted | 82 | 1.06 | 0.96 | 1.18 | 0.25 | 0.67 |
|  |  | *MR Egger* | 82 | 0.92 | 0.70 | 1.20 | 0.54 |  |
|  |  | *Weighted median* | 82 | 0.98 | 0.86 | 1.13 | 0.82 |  |
|  |  | *Weighted mode* | 82 | 0.90 | 0.73 | 1.11 | 0.32 |  |
| Epilepsy | Depression (Als et al. 2023) | Inverse variance weighted | 19 | 0.99 | 0.95 | 1.05 | 0.83 | 0.95 |
|  |  | *MR Egger* | 19 | 0.93 | 0.71 | 1.21 | 0.59 |  |
|  |  | *Weighted median* | 19 | 1.00 | 0.96 | 1.04 | 0.99 |  |
|  |  | *Weighted mode* | 19 | 1.00 | 0.93 | 1.09 | 0.93 |  |
|  | Anxiety (Purves et al. 2022 | Inverse variance weighted | 16 | 0.97 | 0.86 | 1.11 | 0.69 | 1.00 |
|  |  | *MR Egger* | 16 | 1.41 | 0.58 | 3.44 | 0.46 |  |
|  |  | *Weighted median* | 16 | 1.00 | 0.83 | 1.20 | 0.99 |  |
|  |  | *Weighted mode* | 16 | 0.99 | 0.70 | 1.39 | 0.93 |  |
| Childhood obesity | Depression (Als et al. 2023) | Inverse variance weighted | 8 | 1.02 | 0.99 | 1.05 | 0.13 | 0.39 |
|  |  | *MR Egger* | 8 | 1.00 | 0.88 | 1.14 | 0.98 |  |
|  |  | *Weighted median* | 8 | 1.03 | 1.01 | 1.06 | 0.01 |  |
|  |  | *Weighted mode* | 8 | 1.05 | 1.01 | 1.08 | 0.03 |  |
|  | Anxiety (Purves et al. 2022 | Inverse variance weighted | 8 | 1.00 | 0.90 | 1.11 | 0.95 | 1.00 |
|  |  | *MR Egger* | 8 | 0.85 | 0.58 | 1.23 | 0.41 |  |
|  |  | *Weighted median* | 8 | 1.03 | 0.94 | 1.13 | 0.46 |  |
|  |  | *Weighted mode* | 8 | 1.02 | 0.89 | 1.16 | 0.82 |  |

**Table S18** - Results from MR Egger testing for horizontal pleiotropy in the MR analysis between childhood LTCs and anxiety and depression outcomes using the largest adult GWASs (Als *et al.* (2023)) and Purves *et al.* (2020)).

| Exposure | Outcome | Egger intercept | SE | P-value |
| --- | --- | --- | --- | --- |
| Childhood obesity | Depression | 0.00 | 0.01 | 0.72 |
|  | Anxiety | 0.03 | 0.04 | 0.38 |
| Childhood-onset asthma | Depression | 0.00 | 0.00 | 0.96 |
|  | Anxiety | 0.00 | 0.01 | 0.89 |
| Eczema | Depression | 0.00 | 0.01 | 0.50 |
|  | Anxiety | 0.00 | 0.03 | 0.89 |
| Genetic generalised epilepsy | Depression | 0.00 | 0.01 | 0.61 |
|  | Anxiety | -0.02 | 0.03 | 0.43 |
| Juvenile idiopathic arthritis | Depression | 0.01 | 0.01 | 0.17 |
|  | Anxiety* | - | - | - |
| Migraine | Depression | 0.00 | 0.00 | 0.11 |
|  | Anxiety | 0.01 | 0.01 | 0.26 |
| Type 1 diabetes | Depression | 0.00 | 0.00 | 0.29 |
|  | Anxiety | 0.00 | 0.01 | 0.81 |

*Unable to perform test due to only 1 genetic instrument present.

## Supplementary Figures

##
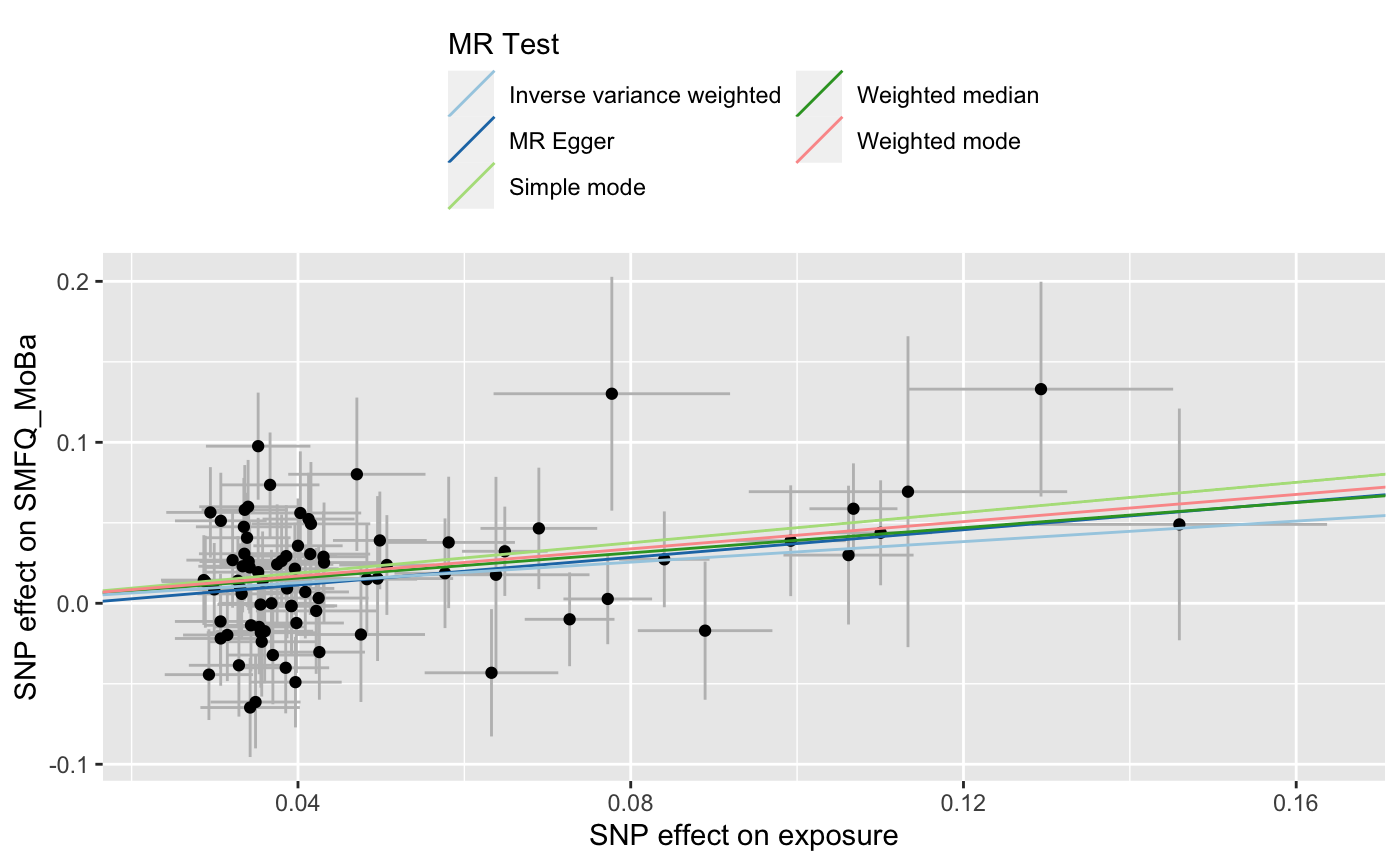


**Figure S1** – MR scatter plot for effect of migraine on elevated depression symptoms at age 14 (self-reported using the SMFQ).

## References

**Als, T. D., Kurki, M. I., Grove, J., Voloudakis, G., Therrien, K., Tasanko, E., . . . Borglum, A. D.** (2023). Depression pathophysiology, risk prediction of recurrence and comorbid psychiatric disorders using genome-wide analyses. *Nat Med* **29**, 1832-1844.

**Bowden, J., Davey Smith, G. & Burgess, S.** (2015). Mendelian randomization with invalid instruments: effect estimation and bias detection through Egger regression. *Int J Epidemiol* **44**, 512-25.

**Bradfield, J. P., Vogelezang, S., Felix, J. F., Chesi, A., Helgeland, O., Horikoshi, M., . . . Grant, S. F. A.** (2019). A trans-ancestral meta-analysis of genome-wide association studies reveals loci associated with childhood obesity. *Hum Mol Genet* **28**, 3327-3338.

**Bulik-Sullivan, B. K., Loh, P. R., Finucane, H. K., Ripke, S., Yang, J., Schizophrenia Working Group of the Psychiatric Genomics, C., . . . Neale, B. M.** (2015). LD Score regression distinguishes confounding from polygenicity in genome-wide association studies. *Nat Genet* **47**, 291-5.

**Bybjerg-Grauholm, J., Pedersen, C. B., Bækvad-Hansen, M., Pedersen, M. G., Adamsen, D., Hansen, C. S., . . . Mortensen, P. B.** (2020). The iPSYCH2015 Case-Cohort sample: updated directions for unravelling genetic and environmental architectures of severe mental disorders. *medRxiv*, 2020.11.30.20237768.

**Ferreira, M. A. R., Mathur, R., Vonk, J. M., Szwajda, A., Brumpton, B., Granell, R., . . . Almqvist, C.** (2019). Genetic Architectures of Childhood- and Adult-Onset Asthma Are Partly Distinct. *Am J Hum Genet* **104**, 665-684.

**Forgetta, V., Manousaki, D., Istomine, R., Ross, S., Tessier, M. C., Marchand, L., . . . Richards, J. B.** (2020). Rare Genetic Variants of Large Effect Influence Risk of Type 1 Diabetes. *Diabetes* **69**, 784-795.

**Hautakangas, H., Winsvold, B. S., Ruotsalainen, S. E., Bjornsdottir, G., Harder, A. V. E., Kogelman, L. J. A., . . . Danish Blood Donor Study Genomic, C.** (2022). Genome-wide analysis of 102,084 migraine cases identifies 123 risk loci and subtype-specific risk alleles. *Nature Genetics* **54**, 152-160.

**Hinks, A., Cobb, J., Marion, M. C., Prahalad, S., Sudman, M., Bowes, J., . . . Thompson, S. D.** (2013). Dense genotyping of immune-related disease regions identifies 14 new susceptibility loci for juvenile idiopathic arthritis. *Nat Genet* **45**, 664-9.

**Hollegaard, M. V., Grove, J., Grauholm, J., Kreiner-Møller, E., Bønnelykke, K., Nørgaard, M., . . . Hougaard, D. M.** (2011). Robustness of genome-wide scanning using archived dried blood spot samples as a DNA source. *BMC Genetics* **12**, 58.

**International League Against Epilepsy Consortium on Complex Epilepsies, Berkovic, S. F., Cavalleri, G. L. & Koeleman, B. P.** (2022). Genome-wide meta-analysis of over 29,000 people with epilepsy reveals 26 loci and subtype-specific genetic architecture. *medRxiv*, 2022.06.08.22276120.

**Lam, M., Awasthi, S., Watson, H. J., Goldstein, J., Panagiotaropoulou, G., Trubetskoy, V., . . . Ripke, S.** (2020). RICOPILI: Rapid Imputation for COnsortias PIpeLIne. *Bioinformatics* **36**, 930-933.

**Mors, O., Perto, G. P. & Mortensen, P. B.** (2011). The Danish Psychiatric Central Research Register. *Scand J Public Health* **39**, 54-7.

**Paternoster, L., Standl, M., Waage, J., Baurecht, H., Hotze, M., Strachan, D. P., . . . Weidinger, S.** (2015). Multi-ancestry genome-wide association study of 21,000 cases and 95,000 controls identifies new risk loci for atopic dermatitis. *Nat Genet* **47**, 1449-1456.

**Pedersen, C. B.** (2011). The Danish Civil Registration System. *Scand J Public Health* **39**, 22-5.

**Pedersen, C. B., Bybjerg-Grauholm, J., Pedersen, M. G., Grove, J., Agerbo, E., Bækvad-Hansen, M., . . . Mortensen, P. B.** (2018). The iPSYCH2012 case–cohort sample: new directions for unravelling genetic and environmental architectures of severe mental disorders. *Molecular Psychiatry* **23**, 6-14.

**Privé, F., Aschard, H., Ziyatdinov, A. & Blum, M. G. B.** (2018). Efficient analysis of large-scale genome-wide data with two R packages: bigstatsr and bigsnpr. *Bioinformatics* **34**, 2781-2787.

**Privé, F., Luu, K., Blum, M. G. B., McGrath, J. J. & Vilhjálmsson, B. J.** (2020). Efficient toolkit implementing best practices for principal component analysis of population genetic data. *Bioinformatics* **36**, 4449-4457.

**Purves, K. L., Coleman, J. R. I., Meier, S. M., Rayner, C., Davis, K. A. S., Cheesman, R., . . . Eley, T. C.** (2020). A major role for common genetic variation in anxiety disorders. *Mol Psychiatry* **25**, 3292-3303.

**Slob, E. A. W. & Burgess, S.** (2020). A comparison of robust Mendelian randomization methods using summary data. *Genet Epidemiol* **44**, 313-329.

**Verbanck, M., Chen, C. Y., Neale, B. & Do, R.** (2018). Detection of widespread horizontal pleiotropy in causal relationships inferred from Mendelian randomization between complex traits and diseases. *Nat Genet* **50**, 693-698.
